# Structural insights into neurokinin 3 receptor activation by endogenous and analogue peptide agonists

**DOI:** 10.1038/s41421-023-00564-w

**Published:** 2023-06-30

**Authors:** Wenjing Sun, Fan Yang, Huanhuan Zhang, Qingning Yuan, Shenglong Ling, Yuanxia Wang, Pei Lv, Zelin Li, Yifan Luo, Dongsheng Liu, Wanchao Yin, Pan Shi, H. Eric Xu, Changlin Tian

**Affiliations:** 1 Department of Endocrinology, Institute of Endocrine and Metabolic Diseases, The First Affiliated Hospital of USTC, School of Life Sciences, Division of Life Sciences and Medicine, Joint Center for Biological Analytical Chemistry, Anhui Engineering Laboratory of Peptide Drug, Anhui Laboratory of Advanced Photonic Science and Technology, University of Science and Technology of China, Hefei, Anhui China; 2 State Key Laboratory of Drug Research, Shanghai Institute of Materia Medica, Chinese Academy of Sciences, Shanghai, China; 3 iHuman Institute, ShanghaiTech University, Shanghai, China; 4 Zhongshan Institute for Drug Discovery, Shanghai Institute of Materia Medica, Chinese Academy of Sciences, Zhongshan, Guangdong China; 5 University of Chinese Academy of Sciences, Beijing, China; 6 School of Life Science and Technology, ShanghaiTech University, Shanghai, China; 7 The Anhui Provincial Key Laboratory of High Magnetic Resonance Image, High Magnetic Field Laboratory, Chinese Academy of Sciences, Hefei, Anhui China

**Keywords:** Cryoelectron microscopy, Hormone receptors

## Abstract

Neurokinin 3 receptor (NK3R) is a tachykinin receptor essential for the hypothalamic-pituitary-gonadal axis. The endogenous peptide agonist neurokinin B (NKB) preferentially activates NK3R, while substance P (SP) binds preferentially to NK1R. In addition, the SP analogue senktide more potently activates NK3R than NKB and SP. However, the mechanisms of preferential binding of peptide and NK3R activation remain elusive. Herein, we determined the cryogenic electron microscopy (cryo-EM) structures of the NK3R–G_q_ complex bound to NKB, SP and senktide. The three NK3R–G_q_/peptide complexes utilize a class of noncanonical receptor activation mechanisms. Combining the structural analysis and functional assay illustrated that the consensus C-termini of the three peptide agonists share a conserved binding mode to NK3R, while the divergent N-termini of the peptides confer the preferential binding of the agonist to NK3R. In addition, the specific interactions between the N-terminus of senktide and the N-terminus and extracellular loops (ECL2 and ECL3) of NK3R lead to the improved activation displayed by senktide compared to SP and NKB. These findings pave the way to understand tachykinin receptor subtype selectivity and provide ideas to rationally develop drugs targeting NK3R.

## Introduction

Neurokinin 3 receptor (NK3R) is widely distributed in different areas of the brain, including the frontal cortex, amygdala, medial septum, and hippocampus^1–3^, and in other organs, such as the ovaries, uterus^4,5^ and those in the digestive system^6–8^. NK3R plays an important role in the hypothalamic-pituitary-gonadal (HPG) axis in humans. When NK3R is dysfunctional, many physiological processes are influenced, including process reward, fluid balance and vasopressin release, cardiovascular function, locomotion, pain, psychiatric conditions, temperature regulation, and reproduction^9–17^. Endogenous neurokinin B (NKB) is the most potent natural agonist of NK3R. It was recently demonstrated that the NKB and NK3R systems are involved in the central control of reproduction^15,18,19^. NKB, kisspeptin, and dynorphin A cooperatively modulate the release of gonadotropin-releasing hormone (GnRH)^20^. In addition, a previous study showed that NKB and NK3R levels are elevated in the placentas of women with preeclampsia. During severe preeclampsia, NK3R expression is upregulated in the endothelial cells of the umbilical vein. Thus, elevated NKB levels may be a diagnostic marker of preeclampsia^21,22^. Considering the important physiological functions of NK3R, this receptor has been a clinical target for schizophrenia, reproductive disorders, hypertension, and preeclampsia treatment^23–26^.

Tachykinins are neuropeptides that are expressed throughout the nervous and immune systems, and as excitatory neurotransmitters, they are important in the regulation of a variety of physiological functions^27^. Among the different tachykinins, substance P (SP), neurokinin A (NKA) and NKB have been well studied. These peptides can function through three different G protein-coupled receptors (GPCRs), neurokinin 1 receptor (NK1R), neurokinin 2 receptor (NK2R), and NK3R^28^, as full agonists with different affinities. NKB, SP, and NKA are 10–11 amino acid linear peptides that share a conserved C-terminal motif, -Phe-Xaa-Gly-Leu-Met-NH_2_, which mediates neurokinin receptor activation, while the divergent N-termini of tachykinin neuropeptides confer the preferential binding to neurokinin receptors^29–31^. NKB preferentially activates NK3R coupled to the Gα_q_ family of G proteins for intracellular signal transduction, while SP and NKA preferentially bind to NK1R and NK2R, respectively^21^. SP can also activate NK3R with lower potency than NKB. The SP analogue senktide (suc-(Asp6, MePhe8) SP (6–11)) is a hexapeptide with an Asp at position 6, a methylated Phe at position 8 and an N-terminus that is protected with succinic acid. Senktide activates NK3R with a selectivity of > 60,000-fold over NK1R and NK2R^14^. In addition, senktide can more potently activate NK3R than the endogenous ligands NKB and SP.

Many studies have been conducted on the selectivity of ligands for tachykinin receptors^31–33^. A message-address model has been proposed for endogenous NKR agonists, dividing the ligand into two functionally distinct parts, the C-terminal region that induces receptor activation (message) and the N-terminal region that provides receptor subtype selectivity (address)^34^. This message-address model is also a popular model for other neuropeptides, such as opioid peptides. Moreover, radioligand binding assays and other pharmacological studies on the chimeric receptor suggested that, together with a minor contribution from the N-terminal portion, the region extending from transmembrane segment II to the second extracellular loop (ECL) plays a role in NKR selectivity^33^.

With the great physiological and pharmacological significance of NK3R, developing NK3R-targeted drugs has attracted increasing attention. To achieve this, it is necessary to clarify the mechanisms of tachykinin signaling that influence diseases, as well as to elucidate the molecular basis of ligand recognition and their diverse activation potencies. However, the detailed interactions that modulate the preferential binding of peptide ligands to NK3R are largely unknown. Here, we used cryogenic electron microscopy (cryo-EM) and functional analysis to gain structural and functional insights into the underlying mechanism by which two endogenous neuropeptides, NKB and SP, and the analogue peptide agonist senktide activate NK3R.

## Results

### Cryo-EM structures of the NK3R–G_q_ complex bound to endogenous (NKB and SP) and analogue (senktide) peptide agonists

A BRIL^35^ fusion protein was introduced at the N-terminus of NK3R, and the C-terminus of the receptor was truncated to improve its expression. To stabilize the binding of NK3R–G_q_ complex to the tachykinin peptides, we applied the NanoBiT^36^ tethering method to the complex assembly (Supplementary Fig. S1a). These modifications were confirmed to have little influence on the pharmacological properties of NK3R (Supplementary Fig. S1b–d). An engineered Gα_q_ chimera^37^ was generated on the basis of the mini-Gα_s/q_71^38^ scaffold, in which the N-terminus was replaced by the corresponding Gα_i1_ sequence. Unless otherwise specified, herein, G_q_ refers to the engineered G_q_ used for structural determination. The engineered G_q_ can bind both Nb35 and scFv16^39,40^, but only the NKB-bound NK3R–G_q_ structure has visible density with both antibodies in the final EM density map. In contrast, the Nb35 subunits could not be built in the SP- and senktide-bound NK3R–G_q_ structures due to the nearly invisible density of the Nb35 components in the final high-resolution maps. The Nb35 components became more visible in lower resolution maps during data processing. This was possible due to the weak occupation of Nb35 or the dynamic characteristics of Nb35 binding to the engineered G_q_ (Supplementary Figs. S2–S4).

**Fig. 1:**
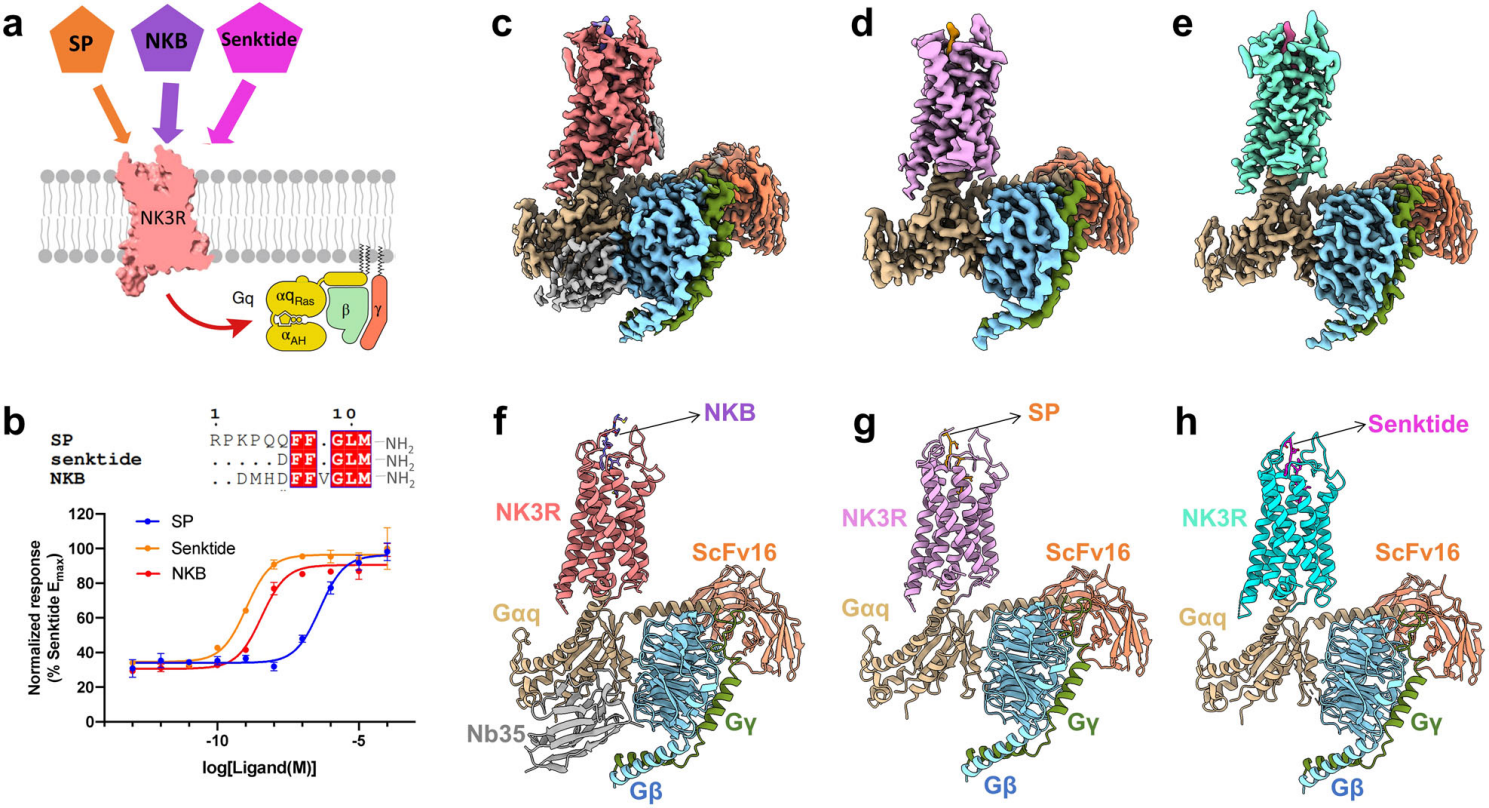
Cryo-EM structure of the tachykinin-bound NK3R–G_q_ complex. **a** Schematic illustration of the affinity and selectivity of tachykinins for NK3R activation and G_q_ protein coupling. **b** IP-one accumulation assay of NK3R activation by NKB, SP, and senktide. Data are expressed as means ± SEM of three independent experiments conducted in triplicate. The sequences of NKB, SP, and senktide are shown. **c**–**e** Cryo-EM density maps of the NKB (**c**)-, SP (**d**)-, and senktide (**e**)-bound NK3R–G_q_ complexes. **f**–**h** Ribbon diagram representation of the cryo-EM structures of the NKB (**f**)-, SP (**g**)-, and senktide (**h**)-bound NK3R–G_q_ complexes colored by subunit (NKB-bound NK3R in salmon, SP-bound NK3R in pink, senktide-bound NK3R in aquamarine, Gα_q_ in tan, Gβ in blue, and Gγ in green).

The cryo-EM structures of the NK3R–G_q_ complexes bound to NKB, SP, and senktide were determined at 2.8 Å, 2.9 Å, and 3.0 Å, respectively (Supplementary Table S1). The resolution of the core region of the complexes could reach 2.5 Å, and the resolution of the ligand-binding pockets could reach ~3.0 Å (Fig. 1c–h; Supplementary Figs. S2–S4). These clear density maps allowed us to unambiguously model most of the components, including tachykinin, the transmembrane domain (TMD), intracellular loops (ICLs) 1–3 and ECLs 1–3, and to illustrate the activation mechanism by the ligands and the conformational changes in NK3R (Supplementary Fig. S5).

### Receptor conformational changes of the NK3R–G_q_ complex upon peptide agonist binding

**Fig. 2:**
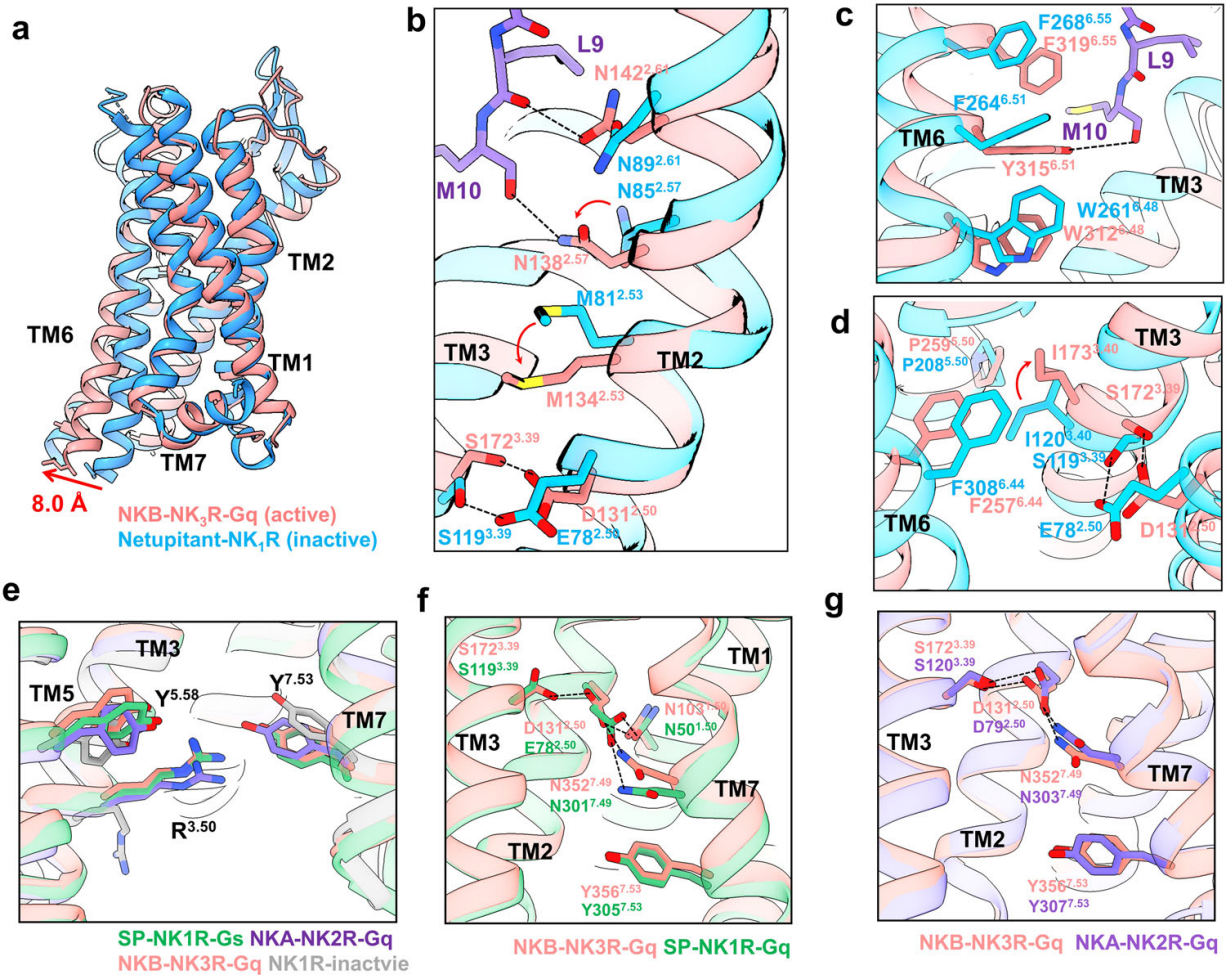
Activation pattern of the tachykinin receptor family. **a** Comparison of the NKB-bound NK3R–G_q_ complex (active) and antagonist netupitant-bound NK1R (inactive) structures. **b** The series of conformational changes in TM2 after NKB binding to NK3R. **c** Conformational changes in the conserved toggle switch motif upon receptor activation. **d** Conformational changes in the conserved PIF motif upon receptor activation. **e** The Y^5.58^–R^3.50^–Y^7.53^ interactions in the SP-bound NK1R, NKA-bound NK2R and NKB-bound NK3R–G_q_ complexes. **f** Comparison of the E78^2.50^–N301^7.49^ interaction in NK1R and the D131^2.50^–N352^7.49^ interaction in NK3R. **g** Comparison of the D79^2.50^–N303^7.49^ interaction in NK2R and the D131^2.50^–N352^7.49^ interaction in NK3R.

The overall conformations of the NK3R–G_q_ complexes bound to the three peptide agonists are highly similar, with root mean square deviation (RMSD) values of 0.53 Å for each entire complex. The structure of the NK3R–G_q_/NKB complex, with higher resolution than the SP- and senktide-bound structures, was applied to analyze NK3R activation. Based on structural comparison of the NK1R–G_q_/SP, NK2R–G_q_/NKA, and NK3R–G_q_/NKB complexes, only very few conformational differences were observed in the intracellular ends of the transmembrane regions (Supplementary Fig. S6). Therefore, structural superimposition of the NK3R–G_q_/NKB complex with netupitant-bound inactive NK1R (PDB: 6HLP) demonstrated that the intracellular end of TM6 in NKB-bound NK3R is shifted outwards by 8.0 Å when measured from the C_α_ carbon of Y184, suggesting that NK3R is in an active state (Fig. 2a). Specifically, a series of conformational changes were also observed after peptide agonist binding to the receptor. After NKB binds to NK3R, the carbonyl oxygen of NKB-Leu9 forms a hydrogen bonding interaction with NK3R-N142^2.61^, where the superscripted number corresponds to Ballesteros-Weinstein numbering^41^ (Fig. 2b). Moreover, the charge interaction between the amide group of NKB-Met10 and NK3R-N138^2.57^ further shifts NK3R TM2 closer to TM3, leading to the conformational change in NK3R-M134^2.53^ and shortening the interaction distance between S172^3.39^ and D131^2.50^ of NK3R (Fig. 2b). At the same time, the amide group in NKB-Met10 can form a stable hydrogen bonding interaction with Y315^6.51^ of NK3R-TM6, which deflects the side chain of NK3R-W312^6.48^ on the toggle switch downwards, and the twist in NK3R-P259^5.50^ causes TM6 to expand outwards. The conformational changes in TM3 and TM6 together affect the deflection of the NK3R-I173^3.40^ and NK3R-F308^6.44^ side chains in the PIF motif and transmit activation signals to the downstream NPxxY motif (Fig. 2c, d).

The interaction between Gα_q_ and NK3R mainly occurs through the α5 helix, which is inserted into the central cavity of the receptor and interacts with the NK3R-TMD and ICLs. Taking NK3R–G_q_/NKB as an example, L384, L388, L393, and V394 in the α5 helix can form a hydrophobic interaction network with L274^5.65^ of TM5 and V297^6.33^ of TM6 (Supplementary Fig. S7). The α5 helix of the G_q_ protein can also form a hydrogen bonding interaction network with NK3R-TM2 and -TM4. Y391 and N392 in the G_q_-α5 helix can form hydrogen bonding interactions with NK3R-N121^2.40^ of TM2 and NK3R-D182^3.49^ of TM3, respectively, and NK3R-R183^3.50^ of TM3 can also interact with the backbone carbonyl oxygen of G_q_-N392. Together, these interactions stabilize G_q_ protein binding and mediate downstream signal transduction.

Unlike most class A GPCRs with resolved structures, NKRs exhibit similar noncanonical activation patterns. The intracellular end of TM7 of NK3R–G_q_ did not move inwards into the central cavity, thus maintaining the inactive conformation. This structural feature has been proposed in previous works on NK1R and NK2R^42,43^. The activation characteristic of the neurokinin receptor family is similar to the noncanonical state of the NTS_1_R–G_i_ complex (PDB: 6OSA^44^), except that the G proteins in the NKR–G_s_/G_q_ complexes were not rotated (Supplementary Fig. S8a). In the canonical activation mode of class A GPCRs (e.g., NTS_1_R), the inwards movement of the intracellular end of TM7 causes Y^7.53^ in the NPxxY motif to shift upwards and away from R^3.50^ (Supplementary Fig. S8a). However, in the NK3R–G_q_/NKB complex, NK3R-R^3.50^ shifts upwards after activation and can form charge interactions with NK3R-Y^7.53^ and NK3R-Y^5.58^, which together stabilize the noncanonically activated state of NK3R (Fig. 2e).

The noncanonical activation patterns between NK1R and NK2R and between NK1R and NK3R are slightly different. NK2R and NK3R retain the highly conserved D^2.50^ (e.g., NK2R-D79^2.50^ or NK3R-D131^2.50^), while NK1R replaces this site with E78^2.50^. In the SP-bound NK1R–G_s_ structure (PDB: 7RMG), NK1R-E78^2.50^ can form a hydrogen bonding interaction with NK1R-N50^1.50^ and a charge interaction with NK1R-N301^7.49^ to stabilize the inactive conformation of the intracellular end of TM7. On the other hand, in the NKB-bound NK3R–G_q_ structure, hydrogen bonding interactions can be formed between NK3R-D131^2.50^ and NK3R-S172^3.39^. The upwards deflection of NK3R-N352^7.49^ allows it to still form a stable charge interaction with NK3R-D131^2.50^ and maintain NK3R-Y356^7.53^ of the NPxxY motif in an inactive state (Fig. 2f, g). Interestingly, the noncanonical activation patterns is conserved for NK3R upon SP or NKB or senktide binding (Supplementary Fig. S8b). In addition, the functional assays suggested that the E78D mutation in NK1R slightly influenced the activating effect of SP (EC_50_ and E_max_), while the D131E mutation in NK3R also had little effect on SP stimulation (Supplementary Fig. S8c). Therefore, either the E^2.50^–N^7.49^ interaction in NK1R or the D^2.50^–N^7.49^ interaction in NK2R and NK3R can maintain the inactive conformational state of the intracellular end of TM7, causing the receptor to exist in a noncanonical activation pattern.

### Specific interactions of the extracellular regions of NK3R with the endogenous peptide agonists NKB and SP

**Fig. 3:**
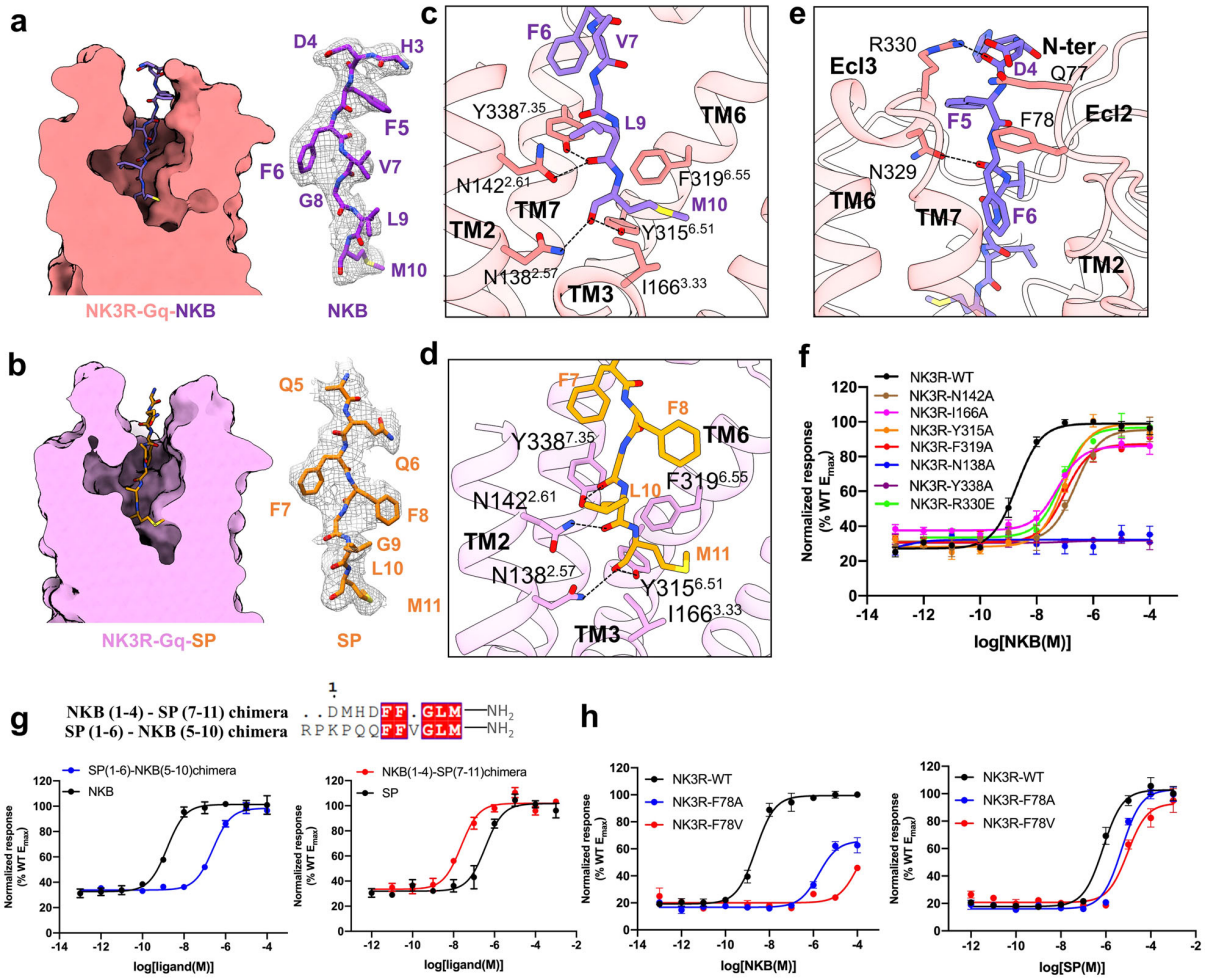
Molecular basis of tachykinin recognition by NK3R. **a**, **b** Space-filling model and cross-sectional view of the NKB-bound (**a**) and SP-bound (**b**) NK3R–G_q_ complexes. The local densities of NKB and SP are shown. **c**, **d** The orthosteric binding pocket of NKB-bound (**c**) and SP-bound (**d**) NK3R. The amino acids involved in the interaction are shown as sticks. **e** The interaction interface of NKB with the N-terminus of NK3R. The interacting amino acids are shown as sticks. **f** IP-one accumulation assays of wild-type NK3R or NK3R with mutations around the ligand-binding pocket to assess activation by NKB. Data are expressed as means ± SEM of three independent experiments conducted in triplicate. **g** IP-one accumulation assays of wild-type NK3R with the NKB and SP chimeras. Data are expressed as means ± SEM of three independent experiments conducted in triplicate. **h** IP-one accumulation assay of wild-type NK3R and NK3R with alanine or valine substitutions at F78 stimulated by NKB and SP. Data are expressed as means ± SEM of three independent experiments conducted in triplicate.

NKB is a high-affinity endogenous full agonist of NK3R. In our structure, the electron microscopy density of NKB can be clearly observed. NKB occupies the orthosteric binding pocket formed by the extracellular domain (ECD) and the TMD of the receptor. NKB shows an overall linear conformation, in which its C-terminus can insert deeply into the NK3R-TMD core (Fig. 3a). In the binding pocket of NK3R, both NK3R-Y338^7.35^ on TM7 and NK3R-N142^2.61^ on TM2 can form hydrogen bonding interactions with the backbone carbonyl oxygen of NKB-Leu9. Moreover, NK3R-N138^2.57^ and NK3R-Y315^6.51^ can interact with the amide group at the C-terminus of the ligand to form a hydrogen bonding network. Moreover, NK3R-F319^6.55^ and NK3R-I166^3.33^ are engaged in hydrophobic interactions with NKB-Met10 (Fig. 3c). Mutations of NK3R-N142^2.61^A, NK3R-I166^3.33^A, NK3R-Y315^6.51^A, and NK3R-F319^6.55^A all caused substantial impacts on NKB-induced NK3R activation. Moreover, the mutations of NK3R-N138^2.57^A and NK3R-Y338^7.35^A abolished the activating effect of NKB on NK3R. Therefore, these residues in the TMD are potentially critical for tachykinin binding or NK3R activation (Fig. 3f; Supplementary Table S2).

Overall, the NK3R–G_q_/SP complex is highly similar to the NK3R–G_q_/NKB complex. From a cross-sectional view of the space-filling models, the orthosteric binding pocket in NK3R–G_q_/SP resembles that of NK3R–G_q_/NKB (Fig. 3a, b). Due to the conserved C-termini of NKB and SP, they share a highly similar binding mode with NK3R (Fig. 3c, d), and this conserved peptide-binding pattern was supported by alanine mutagenesis analysis. Similarly, mutations of N138^7.35^A, N142^2.61^A, Y315^6.51^A, F319^6.55^A, and Y338^7.35^A in NK3R attenuated the potency of NK3R–G_q_ upon SP binding compared to wild-type NK3R as shown by the functional assay (Supplementary Fig. S9a and Table S2).

Notably, although NKB and SP have highly conserved C-termini, these two peptides have divergent N-termini (Fig. 1b), and the N-terminus of NKB has more interactions with NK3R. Specially, N-terminal NK3R-F78 can form π–π interactions with NKB-Phe6 (Fig. 3e; Supplementary Fig. S10). This observation was further supported by a functional assay in which when the N-terminal NK3R-F78 was substituted with alanine or valine, the potency of NKB was substantially attenuated but SP stimulation was only weakly affected (Fig. 3h). In the NK3R–G_q_/SP structure, the N-terminus of the receptor is highly dynamic, and no direct interaction between SP and NK3R was observed. Compared to the binding mode of SP, the interactions between the N-terminus of NKB and NK3R are more stable. We speculated that the interactions between the N-terminus of SP and NK3R contributed to the attenuated potency of SP activation compared to that of NKB. This hypothesis is supported by the fact that replacing the N-terminus of SP with the N-terminus of NKB (SP chimera) led to more potent activation of NK3R than the wild-type SP peptide (Fig. 3g; Supplementary Table S3). In contrast, the NKB chimera with the N-terminus SP substitution showed decreased potency for NK3R activation compared to the wild-type NKB peptide (Fig. 3g; Supplementary Table S3). Moreover, R329 and R330 in ECL3 of NK3R can form hydrogen bonds with the backbone carbonyl groups of NKB-Asp4 and NKB-Phe6, respectively. These results are consistent with previous reports that the divergent N-termini of tachykinin neuropeptides provide certain preferential binding interactions with neurokinin receptors (address). Consequently, the interaction between the N-terminus of NKB and NK3R confers the preferential binding of NKB to NK3R.

### Improved potency of NK3R activation by the senktide versus the endogenous peptide agonist SP

**Fig. 4:**
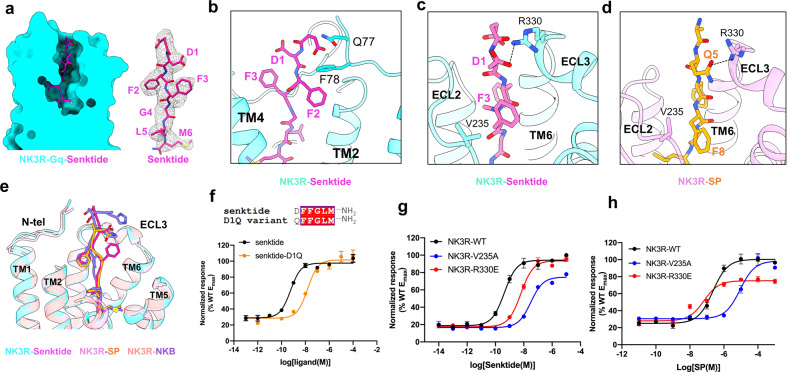
Selective activation of NK3R by senktide. **a** Space-filling model and cross-sectional view of the senktide-bound NK3R–G_q_ complex. The local density of senktide is shown. **b** The interaction interface of senktide and the N-terminus of NK3R. The interacting amino acids are shown as sticks. **c**, **d** Interactions of senktide (**c**) and SP (**d**) with ECL2 and ECL3 of NK3R. **e** Structural comparison of the N-termini of senktide-, NKB-, and SP-bound NK3R–G_q_ complexes. **f** IP-one accumulation assays of wild-type NK3R with the mutant senktide (the first amino acid of senktide was mutated to Q). Data are expressed as means ± SEM of three independent experiments conducted in triplicate. **g**, **h** IP-one accumulation assay of wild-type NK3R and NK3R with mutations of amino acid residues in ECL2 (V235) and ECL3 (R330) with senktide (**g**) and SP (**h**). Data are expressed as means ± SEM of three independent experiments conducted in triplicate.

The SP analogue peptide agonist senktide showed high activation potency and selectivity for NK3R. Compared to the sequence of SP, senktide lacks the 5 amino acids at the N-terminus, and SP-Gln6 is replaced by a succinyl-modified Asp (Fig. 1b). Due to the conserved C-termini of the three peptide agonists, the differences in the N-termini of NKB, SP, and senktide might contribute to their different interactions with the N-terminus or ECLs of NK3R (Fig. 4e). Senktide was observed to sit in an orthosteric binding pocket that is almost identical to those of NKB and SP (Fig. 4a; Supplementary Fig. S9b). The almost identical interactions between the C-termini of senktide, NKB, and NK3R were validated by IP-one accumulation assays (Supplementary Fig. S9c and Table S2). Notably, distinct interactions between the N-terminus of NKB, SP, or senktide and NK3R might account for the improved activation potency and selectivity of senktide versus NKB or SP. First, SP-Gln5 can form a weak hydrogen bonding interaction with NK3R-R330 on ECL3 (Fig. 4d). The N-terminus of senktide is modified by the addition of a succinyl group on D1. The first residue (D1) of senktide was observed to form a stable salt bridge with R330 on ECL3 of NK3R (Fig. 4a, c). The potency of senktide was significantly attenuated with NK3R mutant R330E, but the activation potency of SP was little changed (Fig. 4g, h). Second, senktide-Phe2 was observed to form a hydrophobic interaction with NK3R-F78 (Fig. 4b), which was supported by a functional assay in which substitution of NK3R-F78 with alanine or valine led to attenuated senktide activity (Supplementary Fig. S9d). Third, senktide-Phe3 was observed to engage in hydrophobic interactions with V235 on ECL2 in the senktide-bound NK3R structure (Fig. 4c). Mutating NK3R-V235^ECL2^ to alanine caused a 64-fold decrease in receptor activation upon senktide binding, a 5.2-fold decrease upon NKB binding and a 30-fold decrease upon SP binding, suggesting that there was a greater interaction between senktide-Phe3 and NK3R-ECL2 (Fig. 4g, h). However, in the NK3R–G_q_/SP structure, due to the different orientations of the side chain of SP-Phe8, the distance between SP-Phe8 and NK3R-V235^ECL2^ is longer than that of NK3R–G_q_/senktide complex (Fig. 4d). Collectively, more extensive binding interface areas on ECL2 and ECL3 of NK3R were observed for senktide than SP, and this effect was further manifested by the greater interactions with V235^ECL2^ and R330^ECL3^. Taken together, these data show that the greater interactions between senktide-D1 and NK3R-R330 in ECL3, between senktide-F2 and NK3R-F78, and between senktide-F3 and NK3R-V235 in ECL2 might be responsible for the improved activation potency (address) and selectivity of senktide towards NK3R (signal) compared to those of NKB and SP.

## Discussion

**Fig. 5:**
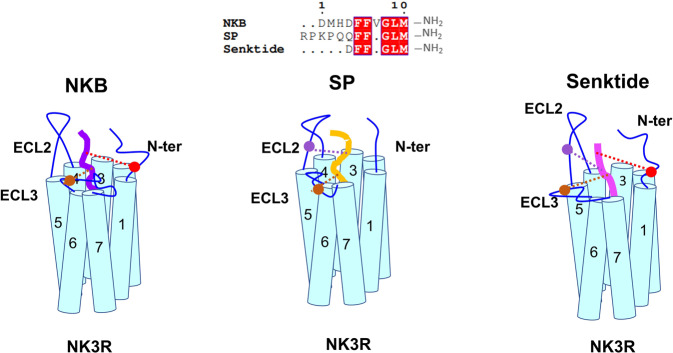
Recognition model of NKB, SP, and senktide bound to NK3R. The interactions between the peptide ligands and NK3R are shown as spheres and dotted lines (red to NK3R’s N-terminus, purple to NK3R’s ECL2, and brown to NK3R’s ECL3). The specific interactions between the N-termini of the ligands and the ECLs of NK3R contribute to the more potent activation by NKB and senktide than by SP.

The cryo-EM structures of the NK3R–G_q_ complex bound to the endogenous peptide agonists NKB and SP and the analogue peptide agonist senktide were determined at resolutions of 2.8 Å, 2.9 Å, and 3.0 Å, respectively. Similar to NK1R and NK2R, NK3R exhibits noncanonical activation patterns in contrast to other class A GPCRs. Previous work reported that endogenous NKR agonists follow a message-address model, in which the C-terminus of the peptide agonist contributes to receptor activation (message) and the N-terminus of the peptide agonist provides receptor subtype selectivity (address). Our structures reveal that the C-terminal consensus sequence (-Phe-Xaa-Gly-Leu-Met-NH_2_) of NKB and SP inserts deeply into the TMD pocket of NK3R and makes highly similar interactions with NK3R. The less conserved N-termini of NKB and SP are responsible for their different potencies and receptor subtype selectivity. In the structure of the NK3R–G_q_/NKB complex, extensive interactions between the N-terminus of NKB and the N-terminal segment of NK3R were observed, while no such interactions were observed for SP (Fig. 5). Swapping the N-termini of NKB and SP suggested that this segment of the peptides plays a specific role in binding specificity for different receptor subtypes. Collectively, the interactions between the N-terminus of NKB and its receptor contribute to the preferential binding of NKB to NK3R.

These structures also reveal the structural basis for NK3R activation by the SP analogue senktide, which shows higher selectivity for NK3R activation. Senktide retains the conserved C-terminus of SP and also has the specific succinylated Asp and methylated Phe. Comparing the structures of NK3R–G_q_/SP and NK3R–G_q_/senktide, the Gln of SP in senktide is substituted with the charged residue Asp of NKB, which maintains the charge interaction between D1 and R330 on ECL3 of NK3R. Substitution of Asp with Gln attenuated the activation potency of senktide (Fig. 4f; Supplementary Table S3), suggesting the essential role of Asp in senktide binding. In addition, F2 of senktide can form a hydrophobic interaction with F78 of NK3R. Moreover, a stronger interaction between senktide-Phe3 and NK3R-V235 in ECL2 was observed. These stable interactions may confer the improved activation potency of senktide. Thus, the specific interactions between the N-terminus of senktide and the N-terminal segment, ECL2 and ECL3 of NK3R result in the improved activation potency of senktide over SP or NKB (Fig. 5).

Notably, site-specific ^19^F-labeled unnatural amino acids were incorporated into NK3R-L232, and ^19^F NMR was performed to further explore the localized conformational rearrangements of NK3R that were induced upon binding SP, NKB, and senktide (Supplementary Fig. S11). Lorentzian deconvolution was used to identify overlapping subpeaks, and different substate distributions (S1, S2, and S3) were observed for the NK3R complex in the apo state. The ^19^F NMR spectra suggested that NK3R existed in a three-state equilibrium between these conformational substates on a slow exchange NMR timescale (Supplementary Fig. S11d). When different peptides bind to NK3R, the ^19^F NMR spectrum displays different spectral line types and component proportions upon conformational rearrangement. Compared to the NK3R-apo state, greater conformational changes were observed when NK3R bound to the more selective peptide agonists NKB and senktide, which is consistent with the different NK3R activation potencies of the three peptides.

In addition to determining the agonist binding selectivity for NK3R, our work also paved the way for discriminating selective agonists according to specific receptor subtype. Despite the high sequence homology between NK1R and NK3R (~70%), different activation modes were observed for the two receptors upon tachykinin binding (Supplementary Fig. S12). Previous studies have shown that both ECL2 and ECL3 of NKRs play important roles in ligand binding^33^. Since some sequence differences are observed in the ECL2 of NK3R and NK1R (Supplementary Fig. S13), ECL2 from NK3R was replaced with that from NK1R for the IP-one accumulation assay. The results demonstrated that the ability of senktide to activate the NK3R/NK1R-ECL2 chimera was abolished in contrast to the full activation of NK3R-WT by this agonist (Supplementary Fig. S13). Therefore, ECL2 of NK3R also participates in preferential interactions with a tachykinin agonist or its analogue.

Taken together, the results from our work here illustrate the detailed activation mechanism of NK3R by the endogenous peptide agonists NKB and SP and the SP analogue senktide, provide important structural templates to understand the mechanism underlying peptide recognition selectivity for NK3R and offer clues for designing selective drug candidates targeting NK3R.

## Materials and methods

### Construct cloning

The optimized coding DNA for wild-type *Homo sapiens* NK3R (UniProt accession: P29371) was synthesized by Genscript. Residues 1–394 of NK3R with an N-terminal thermostabilized apocytochrome b_562_RIL (BRIL)^35^ and a C-terminal LgBiT were cloned into the pFastBac vector using homologous recombination (CloneExpress One Step Cloning Kit, Vazyme). Another TEV cleavage site and the tandem maltose-binding protein tag after LgBiT were added to the NK3R construct to facilitate expression and purification. The engineered Gα_q_ construct was generated on the basis of mini-G_s/q_71^38^ with two dominant-negative mutations (corresponding to G203A and A326S)^37^ to decrease the affinity of nucleotide binding. The N-terminal 1–18 amino acids and the α-helical domain of mini-G_s/q_71 were replaced by the corresponding sequences of human Gα_i1_, providing possible binding sites for three antibody fragments, Nb35, scFv16, and Fab-G50^39,40^. Nb35 and scFv16 were used to stabilize the NK3R–G_q_ complex, and Fab50 was not added in this work. Rat Gβ1 with an N-terminal His6 tag was followed by HiBiT at its C-terminus. The engineered Gα_q_, Gβ1, and bovine Gγ2 were cloned into the pFastBac vector (Invitrogen).

### Expression and purification of Nb35

Nb35^45^ with a C-terminal His6 tag was expressed in *Escherichia coli* BL21 (DE3) and cultured in LB medium with 50 μg/mL ampicillin to an optical density (OD_600_) value of 0.6–1.0 at 37 °C, 180 rpm. IPTG (1 mM) was added to induce Nb35 expression at 27 °C and 180 rpm for 8 h. *E. coli* cells were then collected by centrifugation (4000 rpm, 20 min) and disrupted in 20 mM HEPES, pH 7.4, 100 mM NaCl, 10% glycerol and 1 mM PMSF. Cell pellets were removed by centrifugation (8000 rpm, 30 min), and the supernatant was purified by nickel affinity chromatography (Ni Smart Beads 6FF, Smart Life Sciences). The resin was washed with 30 column volumes of buffer containing 20 mM HEPES, pH 7.4, 100 mM NaCl, 25 mM imidazole and 10% glycerol, and the eluted protein was collected with buffer containing 20 mM HEPES, pH 7.4, 100 mM NaCl, 200 mM imidazole and 10% glycerol. The eluted Nb35 was concentrated and loaded to a HiLoad 16/600 Superdex 75 column (GE Healthcare) preequilibrated with buffer containing 20 mM HEPES, pH 7.4, and 100 mM NaCl. The monomeric fractions were collected and stored with 30% (v/v) glycerol at −80 °C for future use.

### Expression and purification of the NK3R–G_q_ complex

High Five cells were infected with viruses of the receptor (NK3R), Gα_q_, Gβ1, Gγ2, and scFv16 at a ratio of 1:1:1:1:1 for 48 h at 27 °C. The cell pellets were lysed by Dounce homogenization in 20 mM HEPES, pH 7.4, 100 mM NaCl, 10 mM MgCl_2_, 5 mM CaCl_2_, 10% glycerol, EDTA-free protease inhibitor cocktail (TargetMol), and 10 μM peptide (SP, Genscript, NKB/senktide, MedChemExpress). The supernatant was then centrifuged at 30,000 rpm for 30 min to collect the membranes. The washed membranes were resuspended in 20 mM HEPES, pH 7.4, 100 mM NaCl, 10 mM MgCl_2_, 5 mM CaCl_2_, 10% glycerol, 10 μM peptide, 25 mU/mL Apyrase (Sigma-Aldrich), 100 μM TCEP (Sigma-Aldrich), EDTA-free protease inhibitor cocktail and 20 μg/mL Nb35, and incubated at room temperature for 1.5 h. After incubation, 0.5% (w/v) *n*-dodecyl-β-D-maltopyranoside (DDM, Anatrace) and 0.1% (w/v) cholesteryl hemisuccinate (CHS, Anatrace) were used for solubilization at 4 °C for 3 h. The supernatant was collected by centrifugation at 30,000 rpm for 30 min and then incubated with dextrin resin (Dextrin Beads 6FF, Smart Life Sciences) at 4 °C for 3 h. The resin was collected by centrifugation at 500× *g* for 10 min, loaded onto a gravity flow column, and washed with 10 column volumes of buffer containing 20 mM HEPES, pH 7.4, 100 mM NaCl, 10 mM MgCl_2_, 5 mM CaCl_2_, 10% glycerol, 100 μM TCEP, 10 μM peptide, 0.05% (w/v) DDM and 0.01% (w/v) CHS. The detergent of washing buffer was then displaced by 0.1% (w/v) lauryl maltose neopentylglycol (LMNG, Anatrace) and 0.02% (w/v) CHS for 10 column volumes of washing, followed by 0.03% (w/v) LMNG, 0.01% (w/v) glyco-diosgenin (GDN, Anatrace) and 0.008% (w/v) CHS for 20 column volumes of washing. His-tagged TEV protease was then added and incubated with resin at 4 °C overnight. The flow-through was collected and concentrated with an Amicon Ultra Centrifugal Filter (MWCO 100 kDa) and loaded onto a Superdex 200 10/300 GL column (GE Healthcare) with running buffer containing 20 mM HEPES, pH 7.4, 100 mM NaCl, 2 mM MgCl_2_, 100 μM TCEP, 20 μM peptide (SP/NKB/senktide), 0.00075% (w/v) LMNG, 0.00025% (w/v) GDN and 0.0002% (w/v) CHS. The fractions of the monomeric protein complex were collected and concentrated with an Amicon Ultra Centrifugal Filter (MWCO 100 kDa) by 30–50-fold for sample preparation and detection by cryo-EM.

### Cryo-EM grid preparation and data collection

For cryo-EM grid preparation of the NK3R–G_q_/NKB complex, 3 μL of purified protein (3 mg/mL) was loaded onto a freshly plasma-cleaned holey carbon grid (GryoMatrix-M024, R1.2/1.3, 300 mesh, Au) using a Vitrobot chamber (FEI Vitrobot Mark IV). Cryo-EM imaging was performed on a Titan Krios electron microscope at an accelerating voltage of 300 kV using a Gatan K3 Summit direct electron detector with a Gatan energy filter in the Center of Cryo-Electron Microscopy, University of Science and Technology of China (Hefei, China). Micrographs were recorded with a pixel size of 1.07 Å. In total, 5478 movies were obtained at a dose of 55 electrons per Å^2^ for 32 frames. The defocus range of this dataset was –1.2 μm to –2.2 μm.

For cryo-EM grid preparation of the NK3R–G_q_/senktide complex, 3 μL of purified protein (15 mg/mL) was loaded onto a glow-discharged holey carbon grid (Quantifoil, Au300 R1.2/1.3) using a Vitrobot chamber (FEI Vitrobot Mark IV). Cryo-EM images were collected by a Titan Krios G4 at an accelerating voltage of 300 kV equipped with a Faclon IV direct electron detector at the Advanced Center for Electron Microscopy at Shanghai Institute of Materia Medica, Chinese Academy of Sciences. Micrographs were recorded with a pixel size of 0.824 Å. In total, 6520 movies were obtained at a dose of 50 electrons per Å^2^ for 36 frames. The defocus range of this dataset was –1.2 μm to –2.0 μm.

Finally, for cryo-EM grid preparation of the NK3R–G_q_/SP complex, 3 μL of purified protein (10 mg/mL) was loaded onto a glow-discharged holey carbon grid (Quantifoil, Au300 R1.2/1.3) using a Vitrobot chamber (FEI Vitrobot Mark IV). Cryo-EM images were collected by a Titan Krios G4 at accelerating voltage of 300 kV equipped with a Gatan K3 Summit direct electron detector at Advanced Center for Electron Microscopy at Shanghai Institute of Materia Medica, Chinese Academy of Sciences. Micrographs were recorded with a pixel size of 0.824 Å. In total, 5106 movies were obtained at a dose of 50 electrons per Å^2^ for 36 frames. The defocus range of this dataset was –1.2 μm to –2.0 μm.

### Cryo-EM data processing and three-dimensional (3D) reconstruction

Cryo-EM image stacks were aligned using RELION v.3.1.0^46^. Initial contrast transfer function (CTF) fitting was performed with CTFFIND-4.1^47^ from cryoSPARC^48^. For NK3R–G_q_/NKB, the autopicking and two-dimensional (2D) classification were processed using cryoSPARC, producing 2,467,973 particles for further processing. The initial model was reconstructed from a set of particles of 2D classification using the ab initio reconstruction in the first round of 3D classification. With the initial model, two rounds of 3D classifications were carried out. A further round of 3D classification was conducted with a mask on the whole structure, in which 907,878 particles were subjected to 3D autorefinement and polishing. A map with an indicated global resolution of 2.76 Å at a Fourier shell correlation (FSC) of 0.143 was generated from the final 3D refinement and post-processing.

For cryo-EM data of NK3R–G_q_/senktide, autopicking and 2D classification produced 2,848,373 particles for further processing. After 2D classification, all steps were processed using Relion. A map from SP–NK1R–miniG_s/q_70 datasets was used as the initial model and low-pass filtered to 60 Å in the first round of 3D classification. With the initial model, two rounds of 3D classifications were carried out. A further round of 3D classification was conducted with a mask on the receptor, in which 2,457,160 particles were subjected to 3D autorefinement and polishing. A map with an indicated global resolution of 3.0 Å at FSC of 0.143 was generated from the final 3D refinement and subsequently post-processed by DeepEMhancer.

In addition, cryo-EM data of the NK3R–G_q_/SP complexes, autopicking and 2D classification produced 4,102,797 particles for further processing. After 2D classification, all steps were processed using Relion. A map from SP–NK1R–miniG_s/q_70 datasets was used as the initial model and low-pass filtered to 60 Å in the first round of 3D classification. With the initial model, two rounds of 3D classifications were carried out. A further round of 3D classification was conducted with a mask on the receptor, in which 1,980,009 particles were subjected to 3D autorefinement and polishing. A map with an indicated global resolution of 2.93 Å at FSC of 0.143 was generated from the final 3D refinement and subsequently post-processed by DeepEMhancer.

### Model building and refinement

All PDB coordinates predicted by AlphaFold2^48^ served as a starting model for building the atomic model. The G_q_ heterotrimer was built on the basis of the corresponding G protein of the des-Arg^10^-kallidin–B1R–G_q_ (PDB: 7EIB)^49^ complex as a template. All models were fitted into the cryo-EM density map using Chimera^50^ followed by a manual adjustment in Coot^51^. The model was refined by Phenix^52^.

### Expression and purification of NK3R-L232mtfF

For site-specific incorporation of 3′-trifluoromethyl-phenylalanine (mtfF), the NK3R construct mentioned above (with the amber stop mutations at Leu232) was cloned into the pFastBacDual vector.

To increase the insertion efficiency, 2 mM mtfF was added to ESF 921 Insect Cell Culture Medium. High Five cells were infected with viruses of the receptor (NK3R-L232TAG) and another mtfFRS.

The cells were harvested after 48 h at 27 °C and then lysed by Dounce homogenization in 20 mM HEPES, pH 7.4, 100 mM NaCl, 10 mM MgCl_2_, 5 mM CaCl_2_, 10% glycerol, EDTA-free protease inhibitor cocktail, and 10 μM peptide (SP/NKB/senktide). The supernatant was then centrifuged at 30,000 rpm for 30 min to collect the membranes. The washed membranes were resuspended in 20 mM HEPES, pH 7.4, 100 mM NaCl, 10 mM MgCl_2_, 5 mM CaCl_2_, 10% glycerol, 10 μM peptide, 100 μM TCEP, EDTA-free protease inhibitor cocktail, 0.5% (w/v) DDM (Anatrace) and 0.1% (w/v) CHS (Anatrace) for solubilization at 4 °C for 3 h. The supernatant was collected by centrifugation at 30,000 rpm for 30 min and then incubated with dextrin resin (Dextrin Beads 6FF, Smart Life Sciences) at 4 °C for 3 h. The resin was collected by centrifugation at 500× *g* for 10 min, loaded onto a gravity flow column, and washed with 10 column volumes of buffer containing 20 mM HEPES, pH 7.4, 100 mM NaCl, 10 mM MgCl_2_, 5 mM CaCl_2_, 10% glycerol, 100 μM TCEP, 10 μM peptide, 0.05% (w/v) DDM and 0.01% (w/v) CHS. The detergent of washing buffer was then displaced by 0.1% (w/v) LMNG (Anatrace) and 0.02% (w/v) CHS for 10 column volumes of washing, followed by elution with 0.03% (w/v) LMNG, 0.01% (w/v) GDN (Anatrace), 0.008% (w/v) CHS and 10 mM maltose (Diamond) and concentration with an Amicon Ultra Centrifugal Filter (MWCO 100 kDa). Finally, all samples were concentrated to 400 μL after desalting by Zeba Spin Desalting Columns (Thermo Fisher Scientific). Then, 40 μL of D_2_O was added before transferring into 5 mm NMR tubes (Sigma).

### ^19^F-NMR data acquisition and processing

1D ^19^F-NMR spectra were recorded on a Bruker AVANCE III 600 MHz spectrometer equipped with a TCI ^1^H/^19^F-^13^C-^15^N triple resonance cryoprobe in the Experiment Center for Life Science, University of Science and Technology of China (Hefei, China). 1D ^19^F-NMR experiments were recorded at 298 K, with 32,768 scans for the SP experiment, 51,200 scans for the NKB experiment, and 65,536 scans for the senktide experiment. All data were processed with a 40 Hz exponential window function prior to Fourier transformation. For the 1D ^19^F-NMR spectra showing multiple, partially overlapping signal components, signal deconvolution was carried out by using MestReNova.

### IP-one accumulation assay

HEK293T cells were cultured in DMEM (Gibco, Thermo Fisher Scientific) supplemented with 10% FBS and 1% penicillin/streptomycin at 37 °C and 5% CO_2_. The cells were transiently transfected with wild-type or mutant NK3R using Lipofectamine 3000 (Thermo Fisher Scientific). Twenty-four hours post transfection, IP-one experiments were performed using the IP-one assay kit (Cisbio, 62IPAPEC) according to the kit instructions. In brief, cells were harvested and seeded in 384-well plates (7 μL, 13,000 cells per well), and incubated with increasing concentrations of agonist at 37 °C for 1 h. After incubation, 3 μL d2-labeled IP1 and 3 μL cryptate-labeled anti-IP1 antibody (1:20 diluted in lysis and detection buffer) were added to each well. The fluorescence signal was measured by a CLARIOstar microplate reader with excitation at 330 nm and emission at 620 nm and 665 nm. The data were analyzed using GraphPad Prism software 9.0.

### Cell surface expression level analysis

The cell surface expression level was determined by flow cytometry. HEK293T cells were cultured in DMEM (Gibco, Thermo Fisher Scientific) supplemented with 10% FBS at 37 °C and 5% CO_2_. The cells were seeded in six-well plates 24 h before transfection, and then the cells were transiently transfected with wild-type or mutant NK3R using Lipofectamine 3000 (Invitrogen) for 24 h. After transfection, the cells were washed twice with 1 mL of PBS (Gibco, 1000010023) containing 3% BSA. The cells were then pelleted and resuspended in 200 μL PBS containing 3% BSA and 1 μL PE-anti-DYKDDDDK tag (BioLegend, Cat# 637310). After incubation in a 4 °C and dark environment for 30 min, the cells were washed twice with PBS, and the expression levels were detected by flow cytometry (BD Biosciences). All mutant expression levels were normalized to the expression of wild-type NK3R. Each mutant was analyzed in three independent experiments.

### Statistics

Statistical analyses were performed on at least three individual datasets analyzed by GraphPad Prism. Data are means ± SEMs from at least three independent experiments performed in technical triplicate. For the IP-one accumulation assay, data were normalized and analyzed using nonlinear curve fitting for the log (agonist) versus response (three parameters) curves.

## Supplementary information

Supplementary information

## Data Availability

The cryo-EM density maps and corresponding atomic coordinates of the NK3R–G_q_ complex bound with NKB, SP or senktide have been deposited in the Electron Microscopy Data Bank with accession codes EMD-36145, EMD-36146, and EMD-36144, and the Protein Data Bank under accession codes 8JBG, 8JBH, and 8JBF, respectively.
