# Supplementary material for: Structural insights into neurokinin 3 receptor activation by endogenous and analogue peptide agonists
Source: Cell Discov. 2023 Jun 30;9:66. doi: 10.1038/s41421-023-00564-w (PMC10313662; doi:10.1038/s41421-023-00564-w)
Supplement: Supplementary file 1 — Supplementary information [file 41421_2023_564_MOESM1_ESM.pdf]

Supplementary Materials for

Structural Insights into Neurokinin 3 Receptor Activation by Endogenous and Analogue Peptide Agonists

Wenjing Sun[1, #], Fan Yang[1, #], Huanhuan Zhang[1, #], Qingning Yuan[2, #], Shenglong Ling[1], Yuanxia Wang[1], Pei Lv[1], Zelin Li[1], Yifan Luo[1], Dongsheng Liu[3], Wanchao Yin[2,5,6*], Pan Shi[1*], H. Eric Xu[2,6,7 *], Changlin Tian[1,2,4, *]

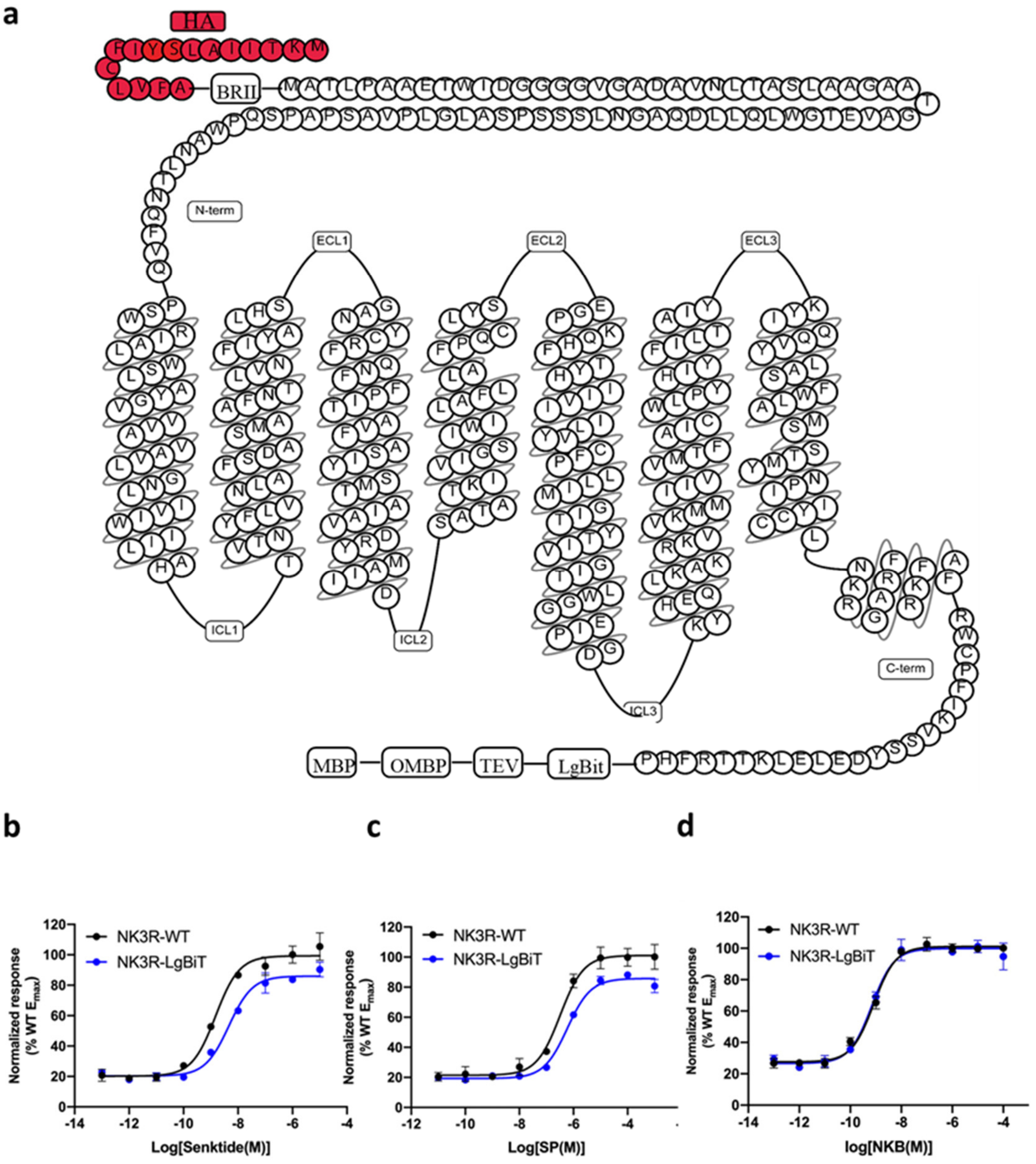

Supplementary Fig. S1 Construct design and functional assay used in this study

a Schematic diagram of NK3R used in the study. The HA signal peptide (red) was linked at the N-terminus.

b, c, d IP-one accumulation assay of wild-type NK3R and NK3R-LgBiT simulation by Senktide, SP and NKB. Data are expressed as the mean ± s.e.m. of three independent experiments conducted in triplicate.

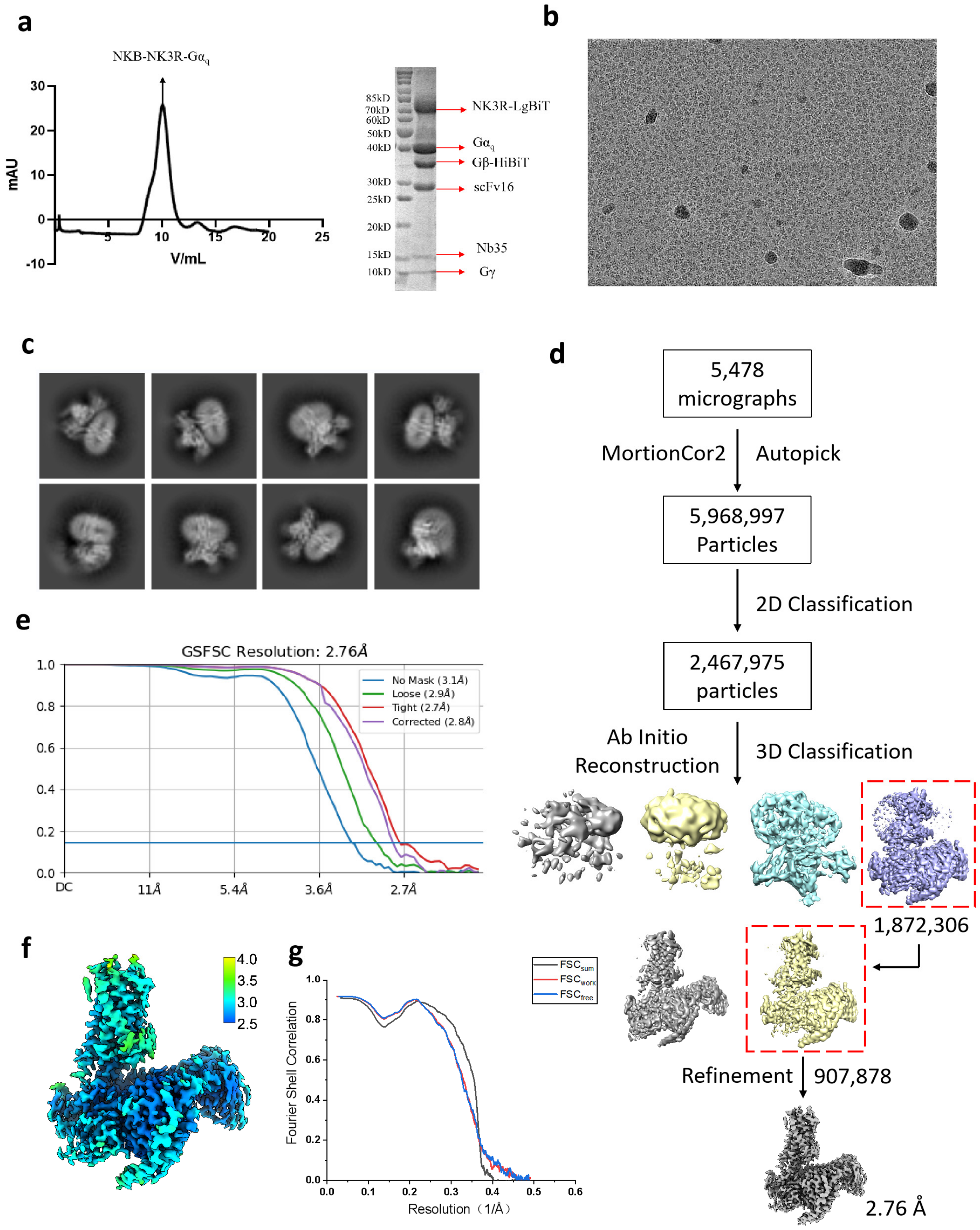

Supplementary Fig. S2 Purification and cryo-EM data processing of the NKB-bound NK3R-G_q complex.

a Size-exclusion chromatography profile and SDS–PAGE analysis of the NK3R-G_q complex bound with NKB. **b** Representative micrograph after motion correction and dose weighting. **c** 2D class averages of the NK3R-G_q complex bound with NKB. **d** Flow chart of cryo-EM data processing using cryoSPARC. **e** Gold-standard FSC validation curves from cryoSPARC. **f** Density map of the NKB-NK3R-G_q complex colored by local resolution estimation. **g** The model-map FSC curves of the NKB-NK3R-G_q complex.

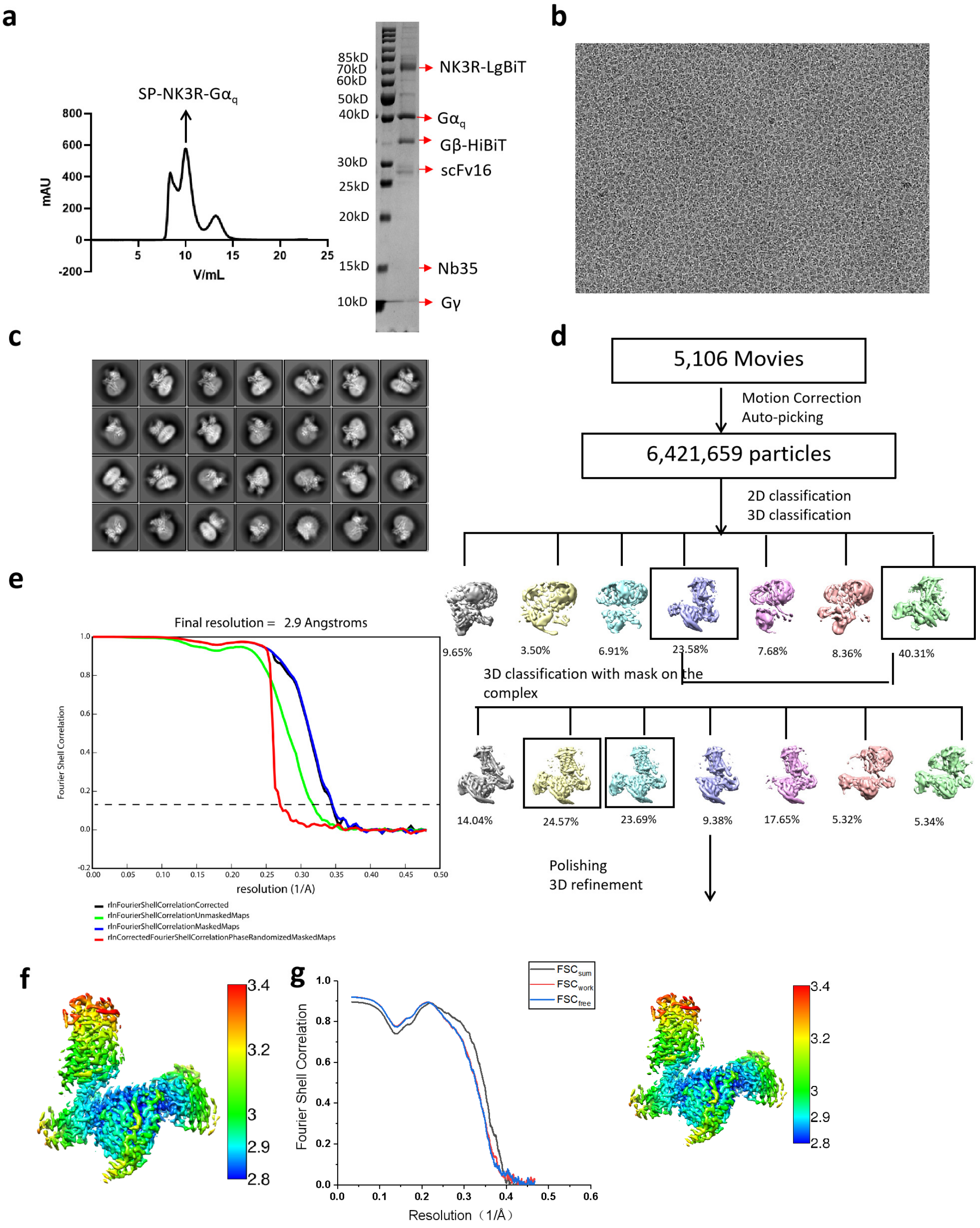

Supplementary Fig. S3 Purification and cryo-EM data processing of the SP-bound NK3R-G_q complex.

a Size-exclusion chromatography profile and SDS–PAGE analysis of the NK3R-G_q complex bound with SP. **b** Representative micrograph after motion correction and dose weighting. **c** 2D class averages of the NK3R-G_q complex bound with SP. **d** Flow chart of cryo-EM data processing using cryoSPARC. **e** Gold-standard FSC validation curves from cryoSPARC. **f** Density map of the SP-NK3R-G_q complex colored by local resolution estimation. **g** The model-map FSC curves of the SP-NK3R-G_q complex.

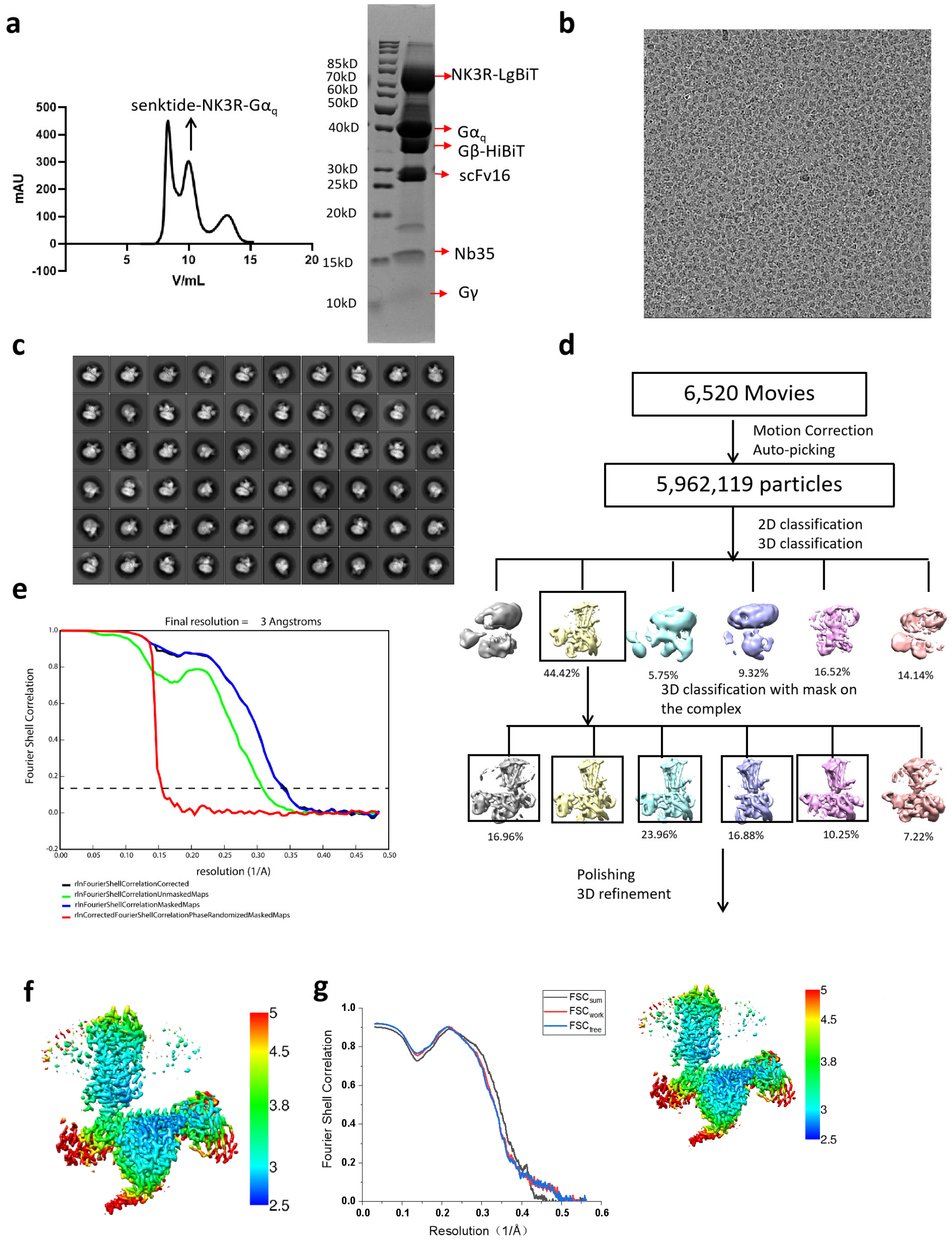

Supplementary Fig. S4 Purification and cryo-EM data processing of the senktide-bound NK3R-G_q complex.

a Size-exclusion chromatography profile and SDS–PAGE analysis of the NK3R-G_q complex bound with senktide. **b** Representative micrograph after motion correction and dose weighting. **c** 2D class averages of the NK3R-G_q complex bound with senktide. **d** Flow chart of cryo-EM data processing using cryoSPARC. **e** Gold-standard FSC validation curves from cryoSPARC. **f** Density map of the senktide-NK3R-G_q complex colored by local resolution estimation. **g** The model-map FSC curves of the senktide-NK3R-G_q complex.

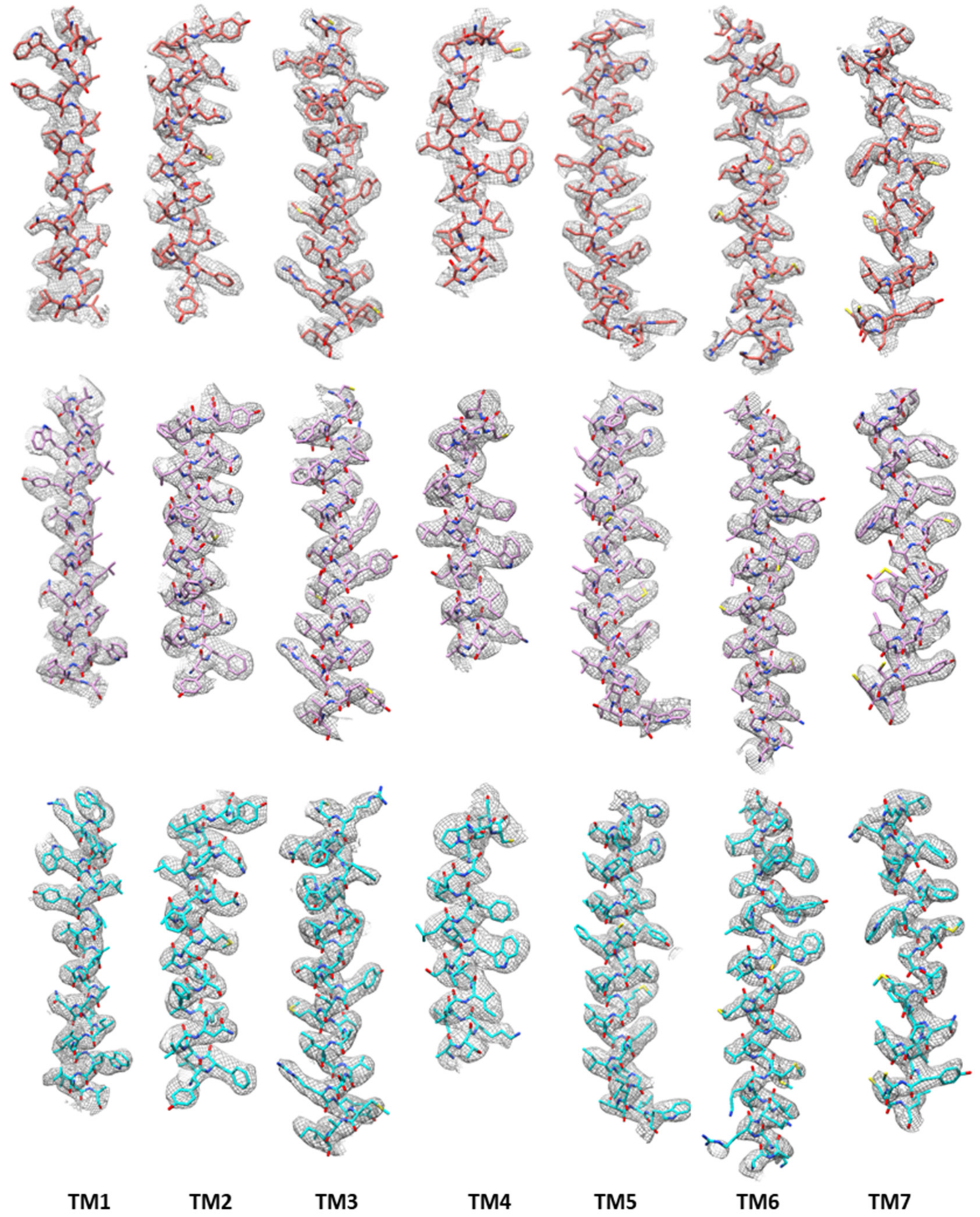

Supplementary Fig. S5 Cryo-EM densities of representative segments of the tachykinin-bound NK3R-G$_q$ complex.

Representative segments of the NK3R-G$_q$ complex bound with NKB, SP and senktide are shown as orange, pink and cyan, respectively.

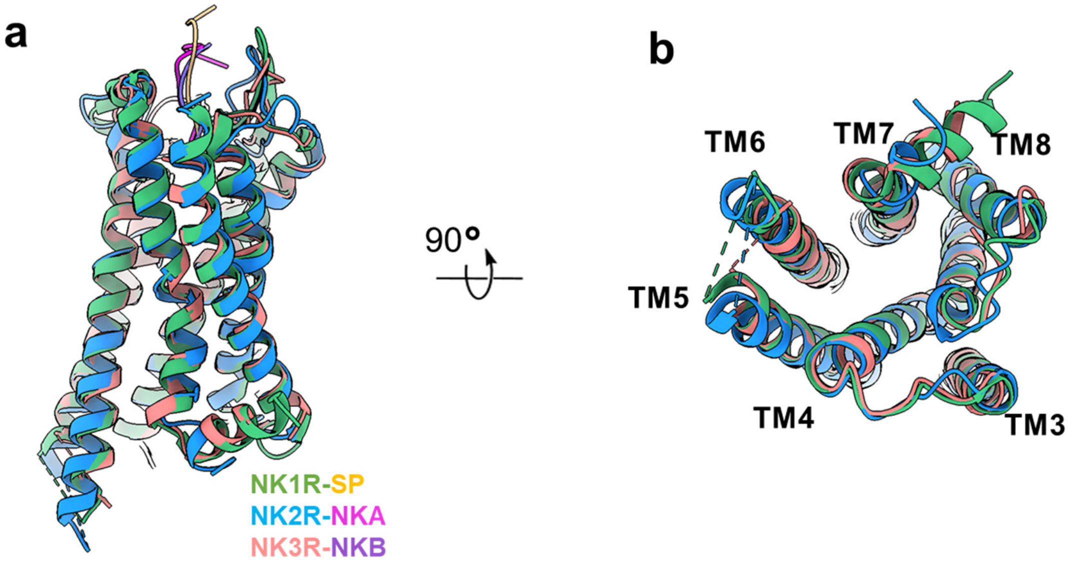

Supplementary Fig. S6 Structural comparison of SP-bound NK1R, NKA-bound NK2R and NKB-bound NK3R-G_q complexes.

Sideview (a) and bottom view (b) of the structural comparison of SP-bound NK1R, NKA-bound NK2R and NKB-bound NK3R-G_q complexes.

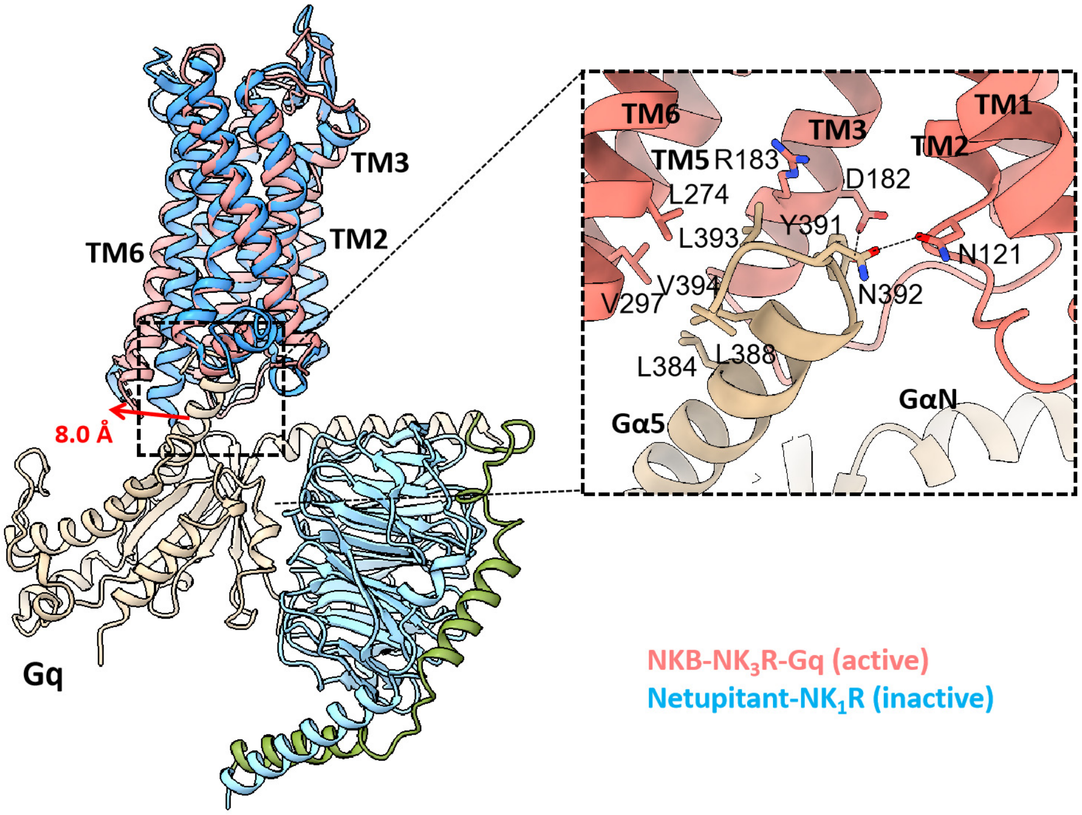

Supplementary Fig. S7 Comparison of the NKB-bound NK3R-G_q complex (active) and antagonist netupitant-bound NK1R (inactive) structures. Interactions between the TMDs of senktide-bound NK3R and G_q protein are enlarged.

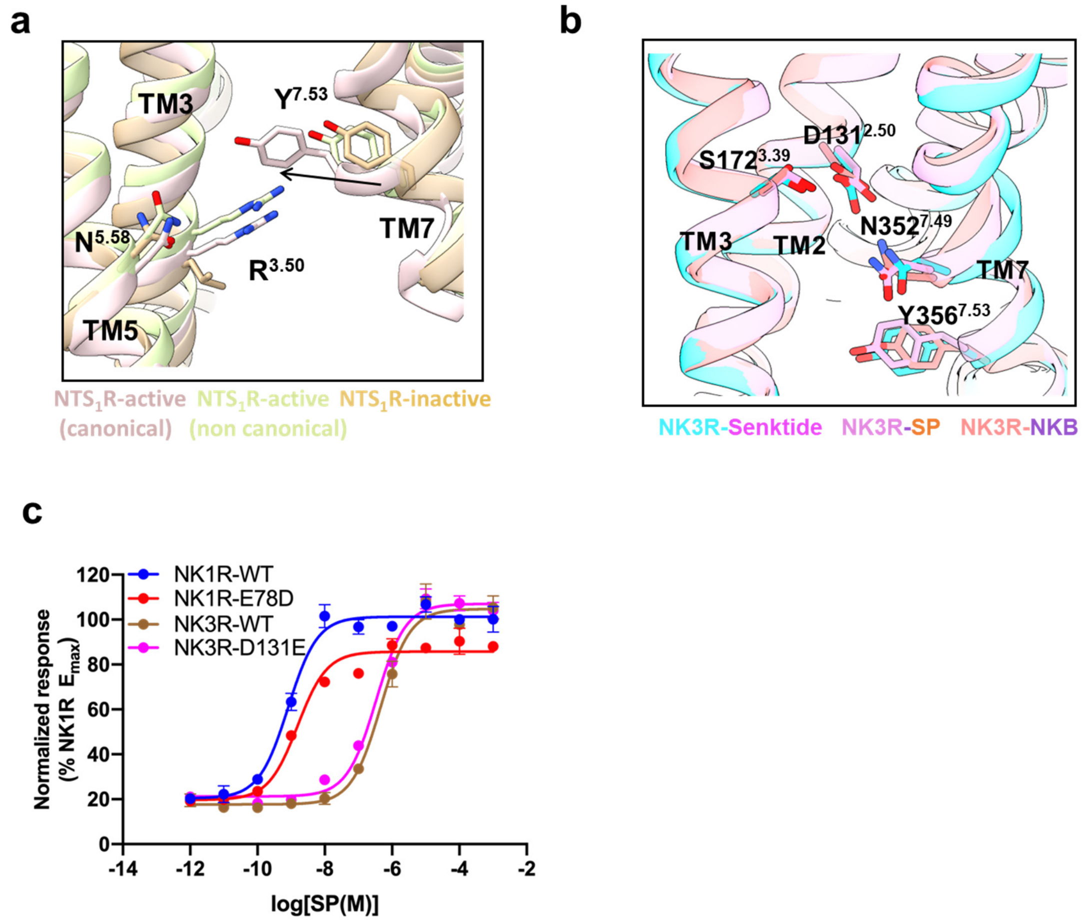

Supplementary Fig. S8 Structural comparison of the canonical and noncanonical active modes of NTS_1R.

a The $N^{5.58}$-$R^{3.50}$-$Y^{7.53}$ interactions of canonical NTS_1R, noncanonical NTS_1R and inactive NTS_1R.

b Comparison of the $D131^{2.50}$-$N352^{7.49}$ interactions of NKB and SP- and senktide-bound NK3R-G_q structures.

c IP-one accumulation assay of aspartic acid substitution of NK1R-E78 and glutamic acid substitution of NK3R-D131 stimulated by SP. Data are expressed as the mean ± s.e.m. of three independent experiments conducted in triplicate.

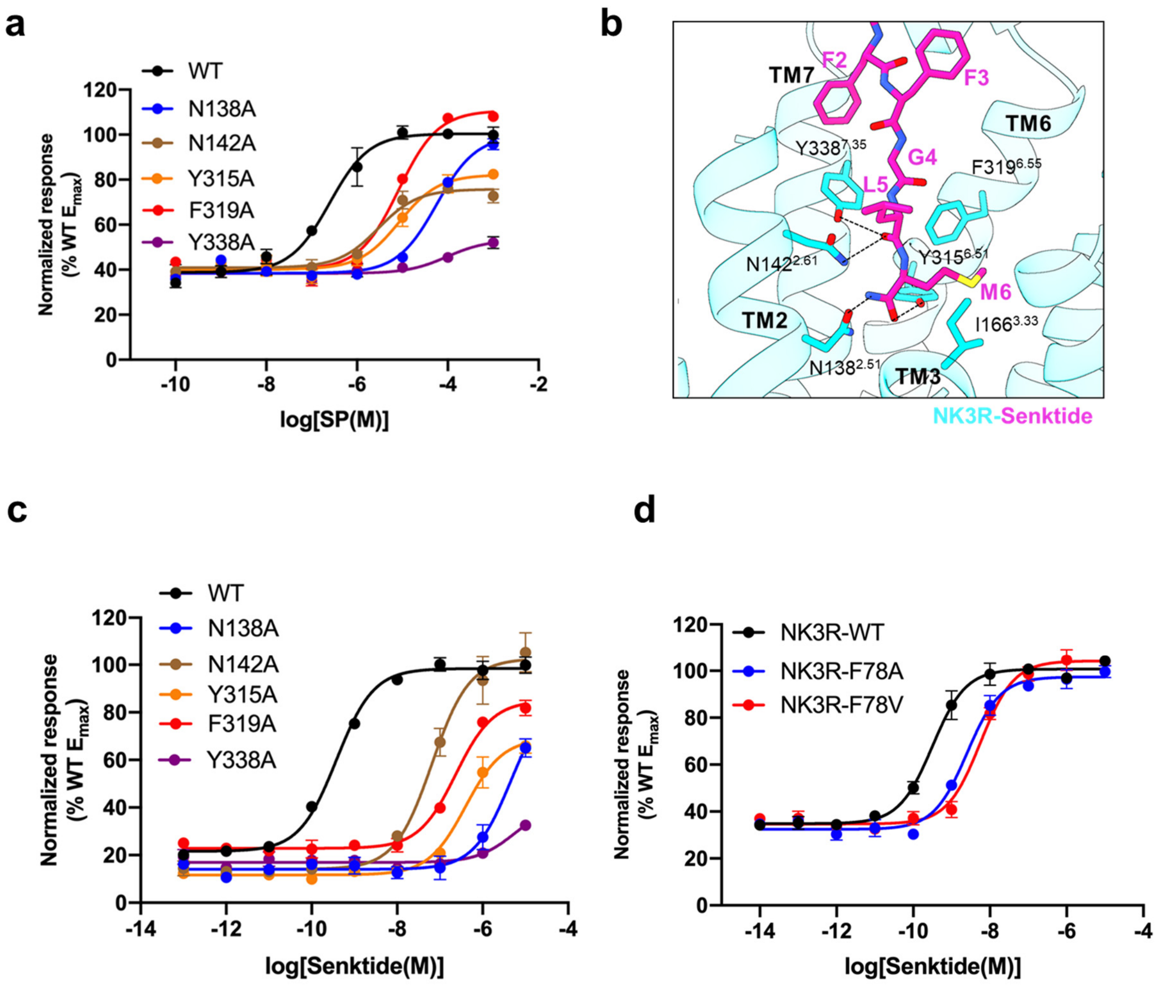

Supplementary Fig. S9 The orthosteric binding pocket of NK3R-G_q-bound SP and senktide.

a IP-one accumulation assay of NK3R activation by SP. Site mutations around the ligand binding pocket disrupt the receptor–ligand interactions, resulting in NK3R malfunction in the IP-one accumulation assay.

b The orthosteric binding pocket of Senktide-bound NK3R. The amino acids involved in the interaction are shown with sticks.

c IP-one accumulation assay of NK3R activation by senktide. Site mutations around the ligand binding pocket disrupt the receptor–ligand interactions, resulting in NK3R malfunction in the IP-one accumulation assay.

d IP-one accumulation assay in which substitution of NK3R-F78 with alanine or valine led to attenuated senktide activity.

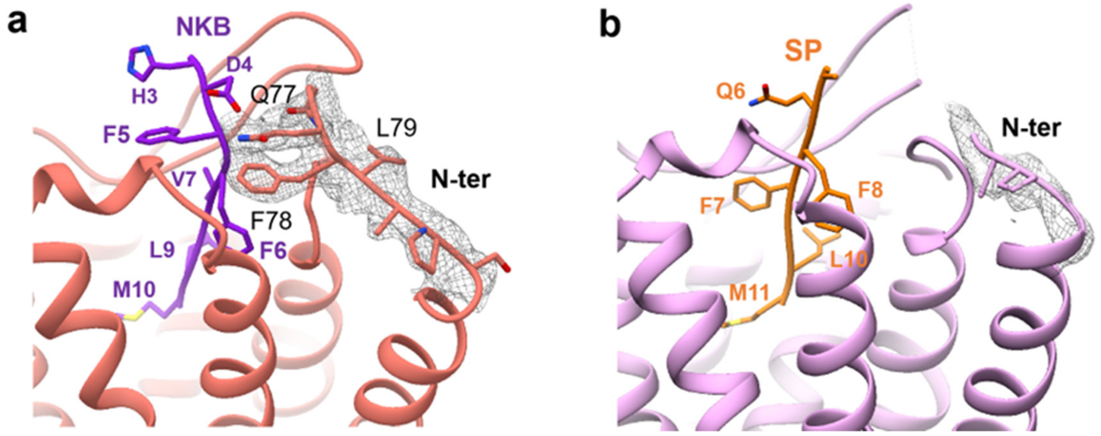

Supplementary Fig. S10 Unsharpened density maps at equivalent enclosed volume thresholds for the N-terminus of NKB-bound (a) and SP-bound (b) NK3R are shown as a mesh.

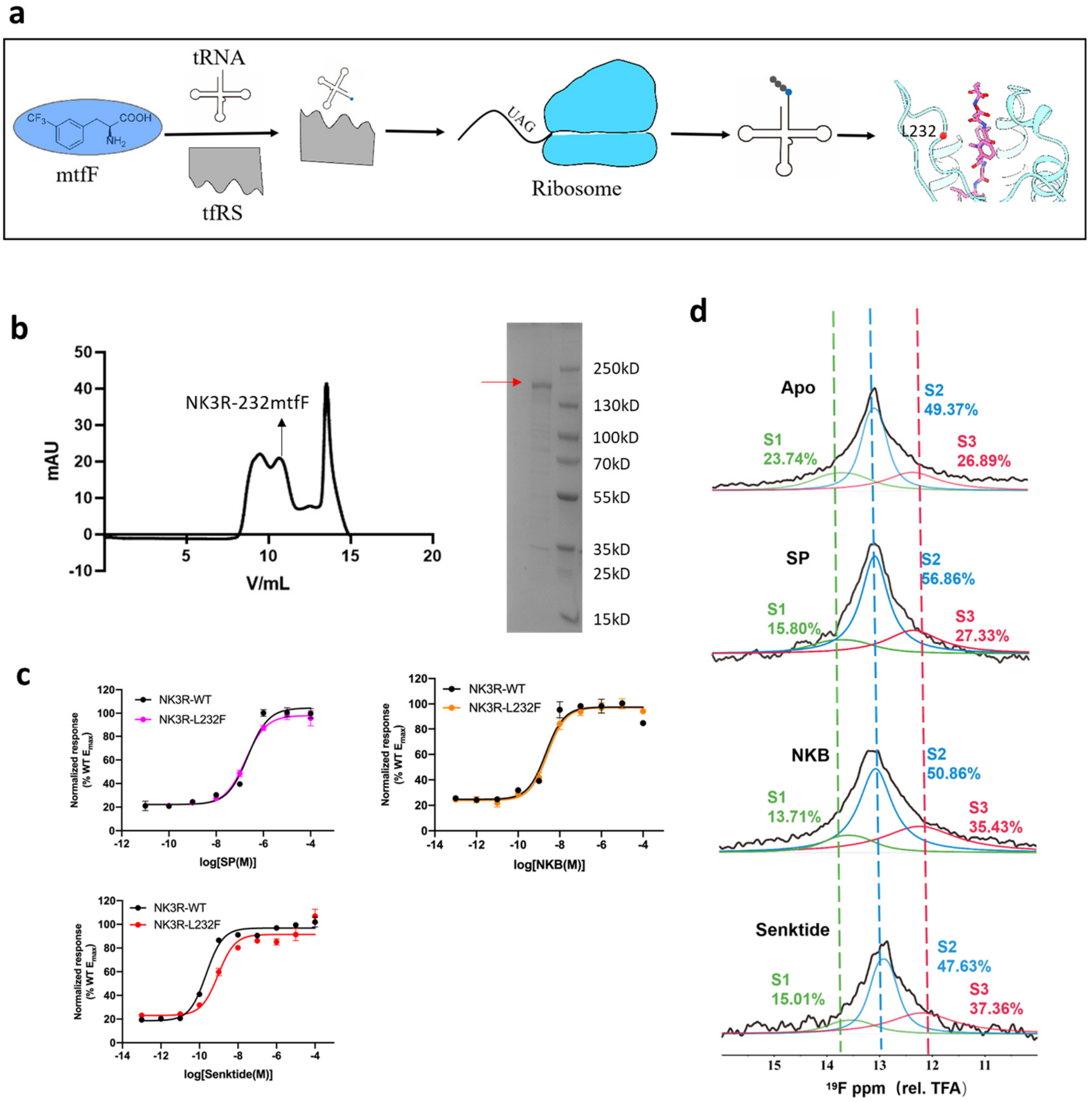

Supplementary Fig. S11 Site-specific ^{19}F-NMR of NK3R-L232-tfmF upon binding of tachykinins.

a Schematic diagram showing the mechanism of site-specific incorporation of tfmF into NK3R-L232.

b Size-exclusion chromatography profile and SDS–PAGE analysis of NK3R-L232-tfmF.

c IP-one accumulation assay shows that NK3R-L232F activation by SP, NKB and senktide is similar to that of wild-type NK3R.

d 1D ^{19}F NMR spectra of NK3R-L232-tfmF with various ligands. The experimental data are shown by black lines, and the components are shown by green, blue and red lines. S1, S2 and S3 represent three states of NK3R-L232-tfmF.

```
                 1        10        20        30        40        50        60
NK3R_HUMAN       MATLPAAETWIDGGGGVGADAVNLTASLAAGAATGAVETGWLQLLDQAGNLSSSPSALGL
NK1R_HUMAN       ..........MDN....................VLPVDS......DLSPNISTNTSEP..
NK2R_HUMAN       .................................MGTCDI......VTEANISSGPESNTT
consensus>70     .....................................#...........N.S........

                          70        80        90       100       110       120
NK3R_HUMAN       PVASPAPSQPWANLTNQFVQPSWRIALWSLAYGVVVAVAVLGNLIVIWIILAHKRMRTVT
NK1R_HUMAN       ...............NQFVQPAWQIVLWAAAYTVIVVTSVVGNVVVMWIILAHKRMRTVT
NK2R_HUMAN       G.............ITAFSMPSWQLALWATAYLALVLVAVTGNAIVIWIILAHRRMRTVT
consensus>70     .................F..P.W...LW..AY...V...V.GN.!V.WIILAH.RMRTVT

                        130       140       150       160       170       180
NK3R_HUMAN       NYFLVNLAFSDASMAAFNTLVNFIYALHSEWYFGANYCRFQNFFPITAVFASIYSMTAIA
NK1R_HUMAN       NYFLVNLAFAEASMAAFNTVVNFTYAVHNEWYYGLFYCKFHNFFPIAAVFASIYSMTAVA
NK2R_HUMAN       NYFIVNLALADLCMAAFNAAFNFVYASHNIWYFGRAFCYFQNLFPITAMFVSIYSMTAIA
consensus>70     NYF.VNLA..#..MAAFN...NF.YA.H..WY%G..%C.F.N.FPI.A.F.SIYSMTA!A

                        190       200       210       220       230       240
NK3R_HUMAN       VDRYMAIIDPLKPRLSATATKIVIGSIWILAFLLAFPQCLYSKTKVMPGRTLCFVQWPEG
NK1R_HUMAN       FDRYMAIIHPLQPRLSATATKVVICVIWVLALLLAFPQGYYSTTETMPSRVVCMIEWPEH
NK2R_HUMAN       ADRYMAIVHPFQPRLSAPSTKAVIAGIWLVALALASPQCFYSTVTMDQGATKCVVAWPED
consensus>70     .DRYMAI!.P..PRLSA..TK.VI..IW..A..LA.PQ..YS..........C.!.WPE.

                          250       260       270       280       290
NK3R_HUMAN       PKQHFT..YHIIVIILVYCFPLLIMGITYTIVGITLWGGEIPGDTCD.KYHEQLKAKRKV
NK1R_HUMAN       PNKIYEKVYHICVTVLIYFLPLLVIGYAYTVVGITLWASEIPGDSSD.RYHEQVSAKRKV
NK2R_HUMAN       SGGKTLLLYHLVVIALIYFLPLAVMFVAYSVIGLTLWRRAVPGHQAHGANLRHLQAMKKF
consensus>70     ........YH..V..L!Y..PL.!....Y.!!G.TLW...!PG............A..K.

                  300       310       320       330       340       350
NK3R_HUMAN       VKMMIIVVMTFAICWLPYHIYFILTAIYQQLNRWKYIQQVYLASFWLAMSSTMYNPIIYC
NK1R_HUMAN       VKMMIVVVCTFAICWLPFHIFFLLPYINPDLYLKKFIQQVYLAIMWLAMSSTMYNPIIYC
NK2R_HUMAN       VKTMVLVVLTFAICWLPYHLYFILGSFQEDIYCHKFIQQVYLALFWLAMSSTMYNPIIYC
consensus>70     VK.M!.VV.TFAICWLP%H.%F.L.....#....K%IQQVYLA..WLAMSSTMYNPIIYC

                  360       370       380       390       400       410
NK3R_HUMAN       CLNKRFRAGFKRAFRWCPFIKVSSYDELELKTTRFHPNRQSSMYTVTRMESMTVVFDPND
NK1R_HUMAN       CLNDRFRLGFKHAFRCCPFISAGDYEGLEMKSTRYLQT.QGSVYKVSRLETTISTVVGAH
NK2R_HUMAN       CLNHRFRSGFRLAFRCCPWVTPTKEDKLELTPTTSLST......RVNRCHTKETLFMAGD
consensus>70     CLN.RFR.GF..AFR.CP.!.....#.LE$..T............V.R............

                  420       430       440       450       460
NK3R_HUMAN       ADTTRSSRKKRATPRDPSFNGCSRRNSKSASATSSFISSPYTSVDEYS
NK1R_HUMAN       EEEPEDGPKATPSSLDLTSNCSSRSDSKTMTESFSFSSNVLS......
NK2R_HUMAN       TAPSEATSGEAGRPQDGSGLWFGYG...LLAPTKTHVEI.........
consensus>70     ...............D................................
```

Supplementary Fig. S12 Sequence alignment of the tachykinin receptor family.

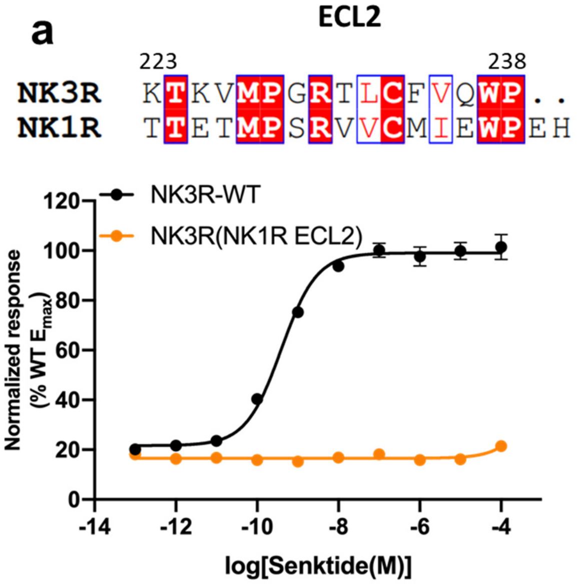

Supplementary Fig. S13 IP-one accumulation assay of wild-type NK3R and NK3R with NK1R-ECL2 chimera simulation by senktide. Sequence alignment of amino acids at ECL2 of NK1R and NK3R (232 to 238) is shown.

Supplementary Table S1| Statistics of cryo-EM data collection, 3D reconstruction and model refinement.

**Data Collection**			
**Protein**	NK3R/NKB	NK3R/SP	NK3R/Senktide
**Microscope**	FEI Titan Krios	FEI Titan Krios	FEI Titan Krios
**Voltage (kV)**	300	300	300
**Detector**	Gatan K3 Summit	Gatan K3 Summit	Gatan K3 Summit
**Detector mode**	Super	Super	Super
**Pixel size (Å)**	0.535	0.535	0.535
**Defocus range (μm)**	-1.2 ~ -2.2	-1.2 ~ -2.2	-1.2 ~ -2.2
**Electron dose (e^-/Å^2)**	55	55	55
**Frames per image**	30	30	30
**Exposure time (s)**	3.5	3.5	3.5
**3D reconstruction**			
**Final Particle number**	907,878	2,457,160	1,980,009
**Symmetry**	C1	C1	C1
**Overall resolution (Å)**	2.76	2.90	3.00
**Model refinement**			
**Model composition**			
**Chains**	6	6	6
**Ligands**	NKB	SP	Senktide
**Nonhydrogen atoms**	8,813	8,915	8,974
**Protein residues**	1,141	1,138	1,140
**Bonds (RMSD)**			
**Length (Å)**	0.009	0.006	0.010
**Angles (°)**	0.880	0.734	0.759
**Ramachandran plot (%)**			

**Outliers**	0.00	0.00	0.09
**Allowed**	5.63	4.22	4.30
**Favored**	94.28	95.78	95.61
**Rotamer outliers (%)**	0.21	8.32	0.10
**MolProbity score**	1.92	2.59	1.85
**Clash score**	10.28	12.05	10.59

Supplementary Table S2 | IP-one assay of wild-type and mutant NK3R and NK1R using NKB, SP or senktide.

Mutant	EC_{50} (nM)	Fold shift	Mean±SEM(n)		Expression (% of WT)
			pEC_{50}	Emax	
**NKB-induced G_q activation of NK3R**					
WT	1.97	1	8.71 ±0.07(11)	100(11)	100(3)
F78A	194.37	98.77	6.71 ±0.48(3)	65.69 ± 1.83(3)	108.68 ± 10.78(3)
F78V	nd	nd	nd	56.33 ± 6.77(3)	100.77 ± 2.84(3)
N138A	nd	nd	nd	42.59 ± 4.06(3)	93.78 ± 17.79(3)
N142A	105.96	53.84	6.97 ±0.20(3)	88.19 ± 3.65(3)	109.36 ± 23.95(3)
I166A	59.18	30.07	7.23 ± 0.02(3)	94.60±4.34(3)	102.62 ± 25.95(3)
Y315A	143.13	72.73	6.84 ±0.10(3)	88.46 ± 2.67(3)	106.47 ± 22.98(3)
V235A	10.32	5.24	7.99 ±0.33(3)	85.65 ± 7.79(3)	94.98 ± 8.45(3)
F319A	55.04	27.97	7.26 ±0.27(3)	90.51 ±0.42(3)	97.06±23.53(3)
Y338A	nd	nd	nd	nd	99.66 ± 9.56(3)
NK3R-lgBiT	1.58	0.80	8.80 ±0.07(3)	99.7 ±0.67(3)	89.33 ± 2.19(3)
L232F	1.14	0.58	8.94±0.18(3)	98.00 ± 3.1(3)	92.25 ± 0.91(3)
**SP-induced G_q activation of NK3R**					
WT	297.00	1	6.53 ±0.02(9)	100(9)	100(9)
F78A	3469.59	11.96	5.46 ±0.09(3)	99.01 ± 2.75(3)	108.68 ± 10.78(3)
F78V	4354.06	15.01	5.36 ±0.16(3)	91.23 ± 2.27(3)	100.77 ± 2.84(3)
D131E	172.50	0.59	6.76 ±0.15(3)	106.33 ± 1.76(3)	101.9 ± 1.92(3)
N138A	912749	3147.41	3.04 ± 1.19(3)	97.88 ± 1.73(3)	93.78 ± 17.79(3)
N142A	3383.77	11.67	5.47±0.04(3)	88.92 ± 15.15(3)	109.36 ± 23.95(3)
Y315A	20126.20	69.40	4.70 ±0.34(3)	89.50 ± 9.55(3)	106.47 ± 22.98(3)
F319A	8386.06	28.92	5.08 ±0.01(3)	102.02 ± 6.95(3)	97.06±23.53(3)
Y338A	nd	nd	nd	nd	99.66 ± 9.56(3)
V235A	8890	30.0	5.05 ±0.21(3)	98.53 ± 4.35(3)	94.98 ± 8.45(3)
R330E	66.8	0.22	7.18 ±0.32(3)	75.21 ± 3.25(3)	105.04 ± 11.19(3)
NK3R-lgBiT	475.30	1.64	6.32 ±0.09(3)	87.30 ± 1.11(3)	89.33 ± 2.19(3)
L232F	125.0	0.43	6.90 ±0.08(3)	99.7 ± 1.2(3)	92.25 ± 0.91(3)
**Senktide-induced G_q activation of NK3R**					
WT	0.36	1	9.44±0.05(3)	100(3)	100(3)
N138A	2362.00	6491.09	5.63 ±0.15(3)	95.48±3.92(3)	93.78 ± 17.79(3)
N142A	28.85	79.25	7.54 ±0.18(3)	394.87 ± 4.04(3)	109.36 ± 23.95(3)
Y315A	115.27	316.68	6.93 ±0.50(3)	81.53 ± 9.42(3)	106.47 ± 22.98(3)
F319A	399.16	1096.66	6.40 ± 0.13(3)	90.87 ± 5.09(3)	97.06±23.53(3)
Y338A	9588	26340.94	5.02±0.13(3)	76.37±17.63(3)	99.66 ± 9.56(3)
N-ter delete	560.41	1539.58	6.25 ± 0.27(3)	54.00 ± 2.52(3)	108.39 ± 5.78(3)
NK3R(NK1R ECL2)	nd	nd	nd	nd	84.16 ± 3.83(3)
V235A	23.30	64.02	7.63 ±0.27(3)	91.90±8.37(3)	94.98 ± 8.45(3)
R330E	2.55	7.02	8.59 ±0.19(3)	105.20 ± 5.25(3)	105.04 ± 11.19(3)
L232F	0.70	1.92	9.15 ± 0.10(3)	94.67 ± 1.45(3)	92.25 ± 0.91(3)

NK3R-lgBiT	3.14	8.64	8.50 ±0.17(3)	93.68 ± 4.09(3)	89.33 ± 2.19(3)
**SP-induced G_q activation of NK1R**					
WT	0.94	1	9.03±0.03(3)	100(3)	100(3)
E78D	4.39	4.68	8.36 ±0.24(3)	99.68±7.07(3)	106.57 ± 2.88(3)

Supplementary Table S3 | IP-one assay of wild-type NK3R using SP, NKB, senktide or their modified forms

**ligand**	**IP1 accumulation**	
	**pEC50±SEM**	**Emax±SEM**
**SP**	6.50 ±0.03(3)	100(3)
**NKB (1-4)-SP (7-11) chimera**	7.59 ±0.06(3)	101 ± 1.85(3)
**NKB**	9.17±0.18(3)	100(3)
**SP (1-6)-NKB (5-10) chimera**	6.27 ±0.17(3)	92.47 ± 3.09(3)
**senktide**	9.63±0.20(3)	100(3)
**senktide-D1Q**	8.39 ±0.29(3)	94.00 ± 3.51(3)
